# A comparison of antimicrobial regimen outcomes and antibiogram development in microbial keratitis: a prospective cohort study in Alexandria, Egypt

**DOI:** 10.1007/s00417-023-06362-0

**Published:** 2024-01-19

**Authors:** Amira A. Nayel, Noha A. Hamdy, Tamer H. Massoud, Nelly M. Mohamed

**Affiliations:** 1 Department of Clinical Pharmacy and Pharmacy Practice, Faculty of Pharmacy, Alexandria University, Alexandria, Egypt; 2 Clinical Pharmacy Department, Alexandria Ophthalmology Hospital, Ministry of Health and Population of Egypt, Alexandria, Egypt; 3 Department of Ophthalmology, Faculty of Medicine, Alexandria University, Alexandria, Egypt; 4 Department of Microbiology and Immunology, Faculty of Pharmacy, Alexandria University, Alexandria, Egypt

**Keywords:** Antibiogram, Antimicrobial resistance, Bacterial keratitis, Corneal ulcers, Empiric therapy, Fungal keratitis, Fortified antibiotics, Microbial keratitis, Time-to-epithelialization, Ulcer healing

## Abstract

**Introduction:**

Antimicrobial resistance in microbial keratitis has not been previously explored in Alexandria. We aim to recommend effective therapies through identification of etiological agents, determination of antimicrobial susceptibilities, and comparing outcomes of empiric topical antimicrobials.

**Methods:**

In this 2022 prospective cohort conducted in Alexandria Main University Hospital cornea clinic, antimicrobial susceptibilities of isolated microorganisms from corneal scrapings were detected and antibiograms were developed. Bacterial (BK), fungal (FK), or mixed fungal/bacterial keratitis (MFBK) patients on empiric regimens were compared for ulcer healing, time-to-epithelialization, best-corrected visual acuity, interventions, and complications.

**Results:**

The prevalent microorganisms in 93 positive-cultures were coagulase-negative staphylococci (CoNS, 30.1%), *Pseudomonas aeruginosa* (14%), and *Aspergillus* spp. (12.9%). CoNS were susceptible to vancomycin (VAN, 100%) and moxifloxacin (MOX, 90.9%). Gram-negative bacteria showed more susceptibility to gatifloxacin (90.9%) than MOX (57.1%), and to gentamicin (GEN, 44.4%) than ceftazidime (CAZ, 11.8%). Methicillin-resistance reached 23.9% among Gram-positive bacteria. Fungi exhibited 10% resistance to voriconazole (VRC). Percentages of healed ulcers in 49 BK patients using GEN + VAN, CAZ + VAN and MOX were 85.7%, 44.4%, and 64.5%, respectively (*p* = 0.259). Their median time-to-epithelialization reached 21, 30, and 30 days, respectively (log-rank *p* = 0.020). In 51 FK patients, more ulcers (88.9%) healed with natamycin (NT) + VRC combination compared to VRC (39.1%) or NT (52.6%) (*p* = 0.036). Their median time-to-epithelialization was 65, 60, and 22 days, respectively (log-rank *p* < 0.001). The VRC group required more interventions (60.9%) than NT + VRC-treated group (11.1%) (*p* = 0.018). In 23 MFBK patients, none healed using NT + CAZ + VAN, while 50% healed using VRC + CAZ + VAN (*p* = 0.052). Regimens had comparable visual outcomes and complications.

**Conclusion:**

Based on the higher detected susceptibility, we recommend empiric MOX in suspected Gram-positive BK, gatifloxacin in Gram-negative BK, and GEN + VAN in severe BK. Due to better outcomes, we recommend NT + VRC in severe FK.

**Trial registration:**

ClinicalTrials.gov identifier, NCT05655689. Registered December 19, 2022– Retrospectively registered, https://clinicaltrials.gov/ct2/show/NCT05655689?cond=NCT05655689.&draw=2&rank=1

**Supplementary Information:**

The online version contains supplementary material available at 10.1007/s00417-023-06362-0.


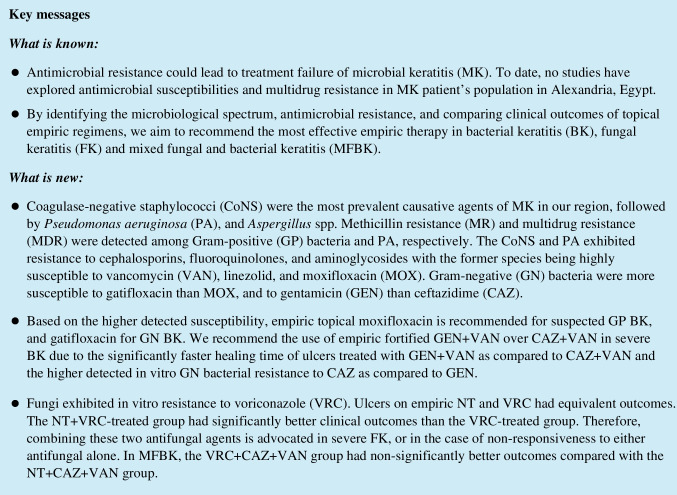


## Introduction

Microbial keratitis (MK) is an ocular emergency caused by an infection of the cornea necessitating the rapid implementation of an effective topical antimicrobial therapy to intercept blindness. The selection of the most efficient topical antimicrobial will largely depend on the correct identification of the infectious agent and the detection of its susceptibility to different antimicrobials to avoid treatment failure [1]. Etiology of MK varies among geographical locations due to differences in climate and patient-related risk factors. In Europe and Middle East, bacteria constitute the most common cause, where coagulase-negative staphylococci (CoNS), *Staphylococcus aureus*, and *Pseudomonas aeruginosa* (PA) are the most prevalent etiological agents of MK, whereas fungi predominate in Asia [1] and Upper Egypt sector [2]. It is documented that CoNS and methicillin-resistant *Staphylococcus aureus* (MRSA) became more resistant to fluoroquinolones [3], and that decreased bacterial susceptibility to moxifloxacin (MOX) has been associated with poor outcomes [4].

Recently, a post hoc analysis of randomized clinical trials in India, reported an increased resistance of fungi to natamycin (NT) and voriconazole (VRC), hence underlying the importance of culture and sensitivity (C&S) testing [5]. The culture of corneal scrapings remains the cornerstone for the diagnosis of causative agents in MK and the subsequent determination of the appropriate antimicrobial therapy. Broad-spectrum empiric regimen is usually initiated after scraping, awaiting the culture results. It is then optimized with selective agents according to the C&S results and the observed clinical response [6]. In general, empiric therapy for BK depends on the administration of MOX or fortified eye drops with either ceftazidime (CAZ) + vancomycin (VAN), or aminoglycoside + VAN. Fortified eye drops are highly concentrated eye drops which are extemporaneously compounded by the reconstitution of parenteral formulations in artificial tears. These fortified eye drops allow the accumulation of high concentrations of antibiotic combinations inside the corneal stroma, an essential prerequisite towards the management of the infection [7]. Cumulative antibiograms, representing the overall antibiotic susceptibility profiles of a certain bacterial species to different antimicrobial agents over one year, are essentially a useful tool for guiding the clinicians to empiric therapy by selecting the antimicrobial to which bacteria are highly susceptible [8].

To date, there is a paucity of information describing the antimicrobial susceptibilities and multidrug resistance in MK in Alexandria. Thus, we conducted this prospective cohort study to identify the prevalence of etiological agents of MK, evaluate their antimicrobial susceptibilities, and compare the clinical outcomes in patients treated with different topical antimicrobial regimens, aiming to recommend the most effective empiric therapy in bacterial keratitis (BK), fungal keratitis (FK), and mixed fungal and bacterial keratitis (MFBK).

## Methods

### Study registration, design, and settings

The study was registered at clinicaltrials.gov (registration number: NCT05655689). It is a prospective cohort conducted in the cornea outpatient clinic located at the Ophthalmology Department in Alexandria Main University Hospital, during a period of one year (from December 2021 to January 2023). The cornea outpatient clinic is a large tertiary eye referral receiving patients from the city of Alexandria and its rural suburbs.

### Study protocol

#### Inclusion criteria

Males and females, aged from 1 to 90 years old, diagnosed with BK, FK, or MFBK during time period from 31st of December 2021 to 1st of January 2023. Culture-positive, culture-negative, and clinically diagnosed cases without cultures were all included.

#### Exclusion criteria

Patients suffering from corneal abrasions, non-infectious ulcers, *Acanthamoeba* and viral keratitis, patients having their files incomplete, non-compliant or lost to follow-up patients, and patients treated with miscellaneous regimens were excluded.

Culture and sensitivity were routinely performed on corneal scrapings in indicated central and large ulcers [6, 7] and the obtained susceptibility results were collected and utilized to develop the antibiogram.

Microbial keratitis patients were treated with empiric antimicrobial eye drops in the case where C&S were not performed or following the corneal scraping procedure and awaiting culture results. Empiric therapy continued if the culture returned negative or was modified when a positive culture result including a susceptible agent was received.

The studied cohorts were BK, FK, and MFBK patients, treated with either one of the following empiric eye drop regimens: MOX 0.5%, fortified (CAZ 5% + VAN 5%), or fortified (GEN 1.4% + VAN 5%) in BK; NT 5%, VRC 1%, or (NT 5% + VRC 1%) in FK; and (NT 5% + CAZ 5% + VAN 5%), or (VRC 1% + CAZ 5% + VAN 5%) in MFBK. Moxifloxacin 0.5% and NT 5% eye drops were commercially available. The preparation of the fortified eye drops, and the methods of their administration are described in supplementary methods. Our study investigators did not recommend or assign any intervention to participants. Adjuvant eye lubricants, cycloplegics, and anti-collagenases (for impending perforation) were prescribed when needed.

Primary outcomes included corneal ulcer healing, time-to-epithelialization, and the antibiogram. Secondary outcomes encompassed improvement in best-corrected visual acuity (BCVA), surgical interventions as therapeutic penetrating keratoplasty (TPK), and complications including corneal perforations, melting, and opacities.

### Antimicrobial Susceptibility Testing (AST)

Corneal scraping specimens and contact lenses (CL) were sent to Alexandria University Medical Microbiology Laboratory for C&S [9]. Bacteria were inoculated on routine and selective culture media then identified microscopically and biochemically (supplementary Figs. S1-S3). The AST was performed using the disc diffusion method in accordance with Clinical and Laboratory Standards Institute (CLSI) guidelines [10]. For each antimicrobial, the percentage susceptibility was calculated by dividing the number of susceptible isolates by the total number of tested isolates and was collected in the cumulative antibiogram. The percentage of intermediate and resistant isolates were grouped as non-susceptible [8]. Culture procedures, AST, definitions of extended-spectrum cephalosporin-resistant *E. coli* (ESCR-*E. coli*) and multidrug-resistant PA (MDR-PA) are described in supplementary file (supplementary methods).

### Risk factors, data collection, and definitions

After clinical diagnosis or culture, the demographics and risk factors (age, gender, living in rural area or working in agriculture, eye trauma either with an object or plant, corneal foreign body (CFB), wearing CL, previous exposure to corticosteroid, past ocular surgery, exposure keratopathy, ocular surface disease (OSD), and systemic risk factors of diabetes or autoimmune diseases) were collected and compared among the following five keratitis groups: Gram-positive bacterial keratitis (GPBK), Gram-negative bacterial keratitis (GNBK), clinically diagnosed bacterial keratitis (CDBK) [11] which included diagnosed cases by clinical signs either after culture-negative results or without cultures, FK, and MFBK. The FK and MFBK groups included culture-positive cases and those diagnosed clinically. Clinical diagnosis was based on the clinical history, as plant trauma and contact lens wear, and the clinical features including elevation of slough, slough texture, well-defined or serrated ulcer margins, infiltrate features (size, depth, and color), presence of satellite lesions, hypopyon, flare, or cells in the anterior chamber [12].

The following parameters were collected from patients’ files, recorded, and compared between empiric regimen groups: disease onset, therapies, clinical picture, baseline clinical characteristics (ulcer/infiltrate size, depth, site, hypopyon, and baseline BCVA), ulcers’ response to therapy, interventions, complications, and outcomes.

Using the slit-lamp biomicroscope caliper, mean ulcer size was calculated as the geometric mean of the longest diameter and the longest perpendicular to this diameter in mm, and classified as small; ≤ 2 mm, moderate; > 2 to 6 mm, and large; ≥ 6 mm [7]. Ulcer depth was classified as superficial, involving 2/3 of the stroma, or deep, involving the posterior 1/3. Ulcer sites were either central, paracentral, or peripheral. Infiltrate size was measured in mm. The presence or absence of hypopyon was recorded. Ulcers were judged as “healed” if complete re-epithelialization with no infiltration was achieved. Healing was confirmed by the absence of fluorescein staining under the slit-lamp biomicroscope using cobalt blue light [13]. Ulcers were considered “not healed” on certain antimicrobial regimens if they had worsened or were not improving, necessitating an intervention as changing the antimicrobial, anterior chamber (AC) wash/ intracameral (IC)/ intrastromal (IS) injections with antimicrobials, or surgery (tectonic/TPK or cyanoacrylate corneal gluing) [14]. The time-to-epithelialization (ulcer healing time) was recorded. It was defined as the time, in days, required for re-epithelialization after starting treatment (or from diagnosis). The response to antimicrobials was monitored every 72 h and the ulcer or infiltrate size response was classified as “improving” if the ulcer/infiltrate’s size was decreasing, “worse” if it was increasing or in the case of hypopyon appearance, and “not changing” if it remained stationary [15]. The BCVA was recorded as decimal values of Snellen fractions on the first visit prior to treatment (baseline BCVA) and on the last visit after ulcer healing (post-treatment BCVA). The improvement in BCVA was calculated by detecting the difference between post-treatment and baseline BCVA. Values smaller than 0.02 were described by the semi-quantitative vision of counting fingers (CF), hand motion (HM), light perception (PL), and no light perception (NPL). These were quantified by their conversion into the equivalent decimal values using the Freiburg Visual Acuity Test (FrACT) as follows: the mean value of CF was 0.014, which is the mean of Snellen fractions ranging from 0.01 to 0.02, while the mean value of HM was 0.0052, which is the mean of Snellen fractions ranging from 0.0033 to 0.0090. Light perception and NPL vision were given the values of 0.0016 and 0.0010, respectively [16].

Complications including corneal melting, opacities, anterior synechiae, adherent leukoma, descemetocele, staphylomas, atrophy, and corneal perforation were assessed under slit-lamp biomicroscope. The latter was assessed by the Seidel test under cobalt blue light. Endophthalmitis was assessed by ultrasound.

### Sample size calculation and statistical analysis

The sample size was calculated based on the assumption of logistic regression using epiinfo v. 3 opensource calculator-SS cohort [17]. To detect an effect size of 30% difference in healing rate or higher, 50 patients were allocated to three treatment groups, and 24 patients to two groups, with two-sided significance levels of 5% and 80% power. Data was analyzed using IBM SPSS statistics for Windows, v. 20.0. Armonk, NY: IBM Corp. Pearson’s Chi-square test, Fisher’s exact test, or Monte Carlo correction were used for nominal or categorical data. Quantitative variables were compared using Student’s t-test, ANOVA F-test, and Post hoc test (LSD) for normally distributed, and the Mann–Whitney *U*- test, Kruskal Wallis test, and Post hoc Dunn's multiple comparisons test for abnormally distributed variables.

## Results

**Fig. 1:**
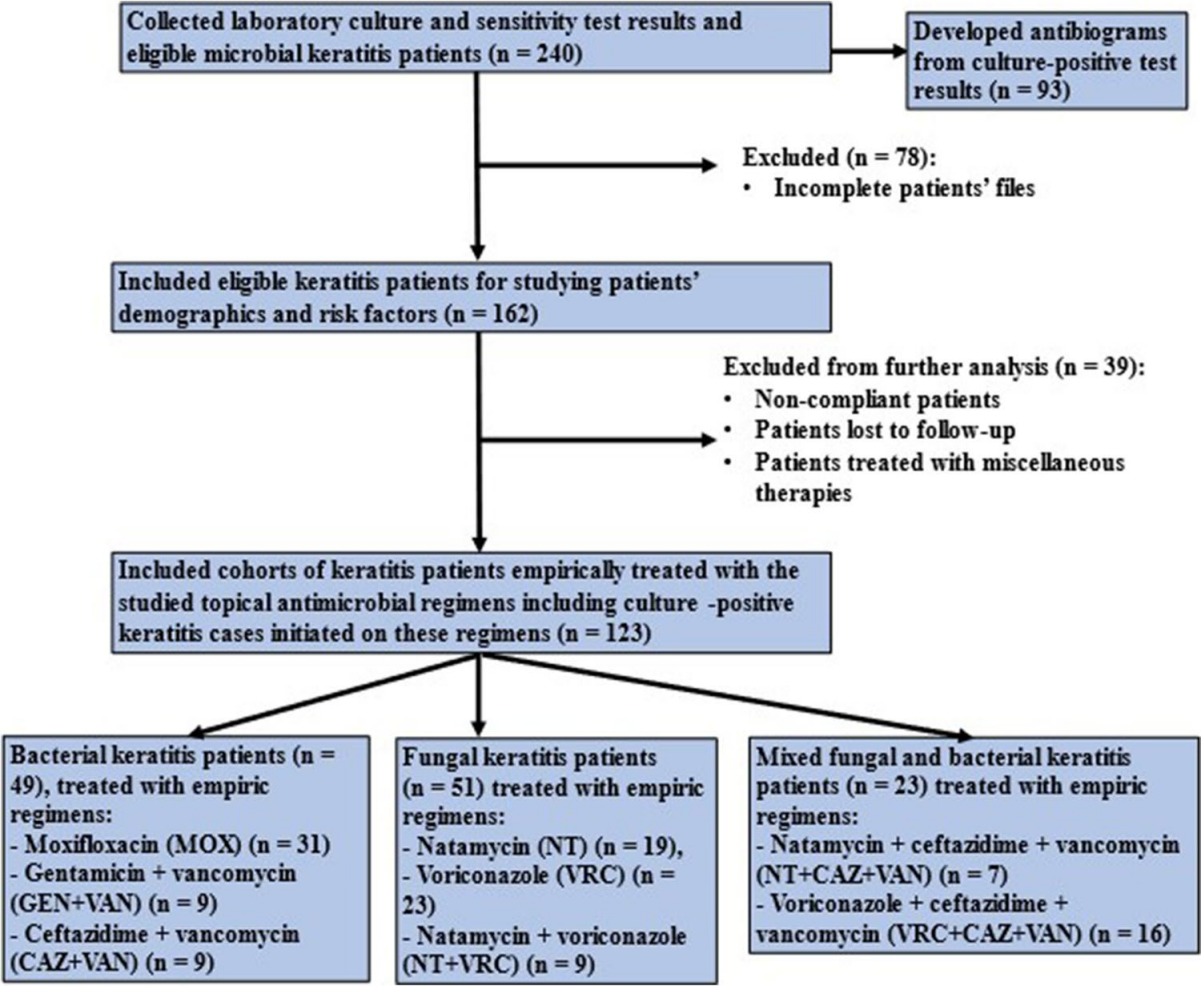
A flow diagram for the study profile

During the study period, 240 eligible keratitis patients were recruited, and their C&S results were revised. Out of these 240 patients, 93 had positive cultures which were used for the development of antibiogram. Incomplete patient profiles were excluded. We included 162 corneal ulcer patients, and their demographics and risk factors were studied. After excluding 39 patients, 123 were observed for treatment outcomes of the studied topical empiric regimens (Fig. 1).

The demographics and risk factors within the different keratitis groups (*n* = 162) are described in Table 1. The age of the patients ranged from 1 to 81 years old. The mean age, in years, was in the fourth decade, except in the GPBK group where patients were younger (37.2 ± 20.14) (*p* = 0.044). Living in rural area or working in agriculture (28.1%, *p* = 0.014) and male gender (68.4%, *p* = 0.003) were significantly associated with FK. Females, however, prevailed in GNBK (76.5%, *p* = 0.003). The most common risk factor was eye trauma (28.4%) (46/162), either by an object (14.2%) or plant (14.2%), followed by living/working in agricultural area (16%), then wearing CL (13.6%). Vegetative eye trauma (28.1%, *p* = 0.004), CFB (21.6%, *p* = 0.027), and exposure keratopathy (8.3%, *p* = 0.030) were significantly associated with FK, CDBK, and GPBK, respectively. In culture-positive BK (GPBK and GNBK), non-CL keratitis represented 66% (27/41), while 34% were CL wearers. Wearing CL was significantly associated with GNBK (52.9%, *p* < 0.001). Other investigated risk factors were non-significant.

**Table 1:** Comparison of demographics and risk factors in the different keratitis groups

Demographics and risk factors	GPBK (*n* = 24)	GNBK (*n* = 17)	CDBK (*n* = 37)	FK (*n* = 57)	MFBK (*n* = 27)	*p-*value
	*N* (%)	*N* (%)	*N* (%)	*N* (%)	*N* (%)	
Age (years)						
< 10 (Children)	2 (8.3)	0 (0)	1 (2.7)	2 (3.5)	1 (3.7)	0.734
10– < 18 (Adolescents)	1 (4.2)	2 (11.8)	1 (2.7)	2 (3.5)	0 (0)	
18– 59 (Adults)	16 (66.7)	12 (70.6)	24 (64.9)	36 (63.2)	15 (55.6)	
≥ 60 (Elderly)	5 (20.8)	3 (17.6)	11 (29.7)	17 (29.8)	11 (40.7)	
Min. – Max	1 – 72	16 – 65	7 – 80	5 – 81	5 – 75	**0.044**
Mean ± SD	37.25^b^ ± 20.14	39.59^a^ ± 18.22	45.57^ab^ ± 18.54	48.35^a^ ± 16.19	49.63^a^ ± 17.11	
Median (IQR)	37 (23 – 52)	36 (22 – 58)	41 (35 – 60)	50 (40 – 60)	51 (36 – 64.5)	
Sex						
Male	12^ab^ (50.0)	4^b^ (23.5)	25^a^ (67.6)	39^a^ (68.4)	11^ab^ (40.7)	**0.003**
Female	12^ab^ (50.0)	13^b^ (76.5)	12^a^ (32.4)	18^a^ (31.6)	16^ab^ (59.3)	
Living in rural area /working in agriculture	2^ab^ (8.3)	2^ab^ (11.8)	1^b^ (2.7)	16^a^ (28.1)	5^a^ (18.5)	**0.014**
Trauma with an object	7 (29.2)	1 (5.9)	5 (13.5)	5 (8.8)	5 (18.5)	0.153
Vegetative trauma	1^ab^ (4.2)	2^ab^ (11.8)	1^a^ (2.7)	16^b^ (28.1)	3^ab^ (11.1)	**0.004**
Corneal foreign body	1^abc^ (4.2)	0^c^ (0)	8^b^ (21.6)	3^ac^ (5.3)	5^abc^ (18.5)	**0.027**
Contact lens wear	5^ab^ (20.8)	9^b^ (52.9)	5^a^ (13.5)	3^a^ (5.3)	5^a^ (18.5)	** < 0.001**
Prior corticosteroids	2 (8.3)	2 (11.8)	2 (5.4)	5 (8.8)	2 (7.4)	0.910
Past ocular surgery						
No	20 (83.3)	13 (76.5)	34 (91.9)	49 (86)	18 (66.7)	0.104
Yes	4 (16.7)	4 (23.5)	3 (8.1)	8 (14.0)	9 (33.3)	
Cataract	1 (25.0)	2 (50.0)	1 (33.3)	1 (12.5)	2 (22.2)	0.579
TPK	2 (50.0)	2 (50.0)	1 (33.3)	7 (87.5)	5 (55.6)	
LASIK	1 (25.0)	0 (0.0)	0 (0.0)	0 (0.0)	0 (0.0)	
Pterygium	0 (0.0)	0 (0.0)	0 (0.0)	0 (0.0)	1 (11.1)	
Others	0 (0.0)	0 (0.0)	1 (33.3)	0 (0.0)	1 (11.1)	
Ocular surface disease	1 (4.2)	1 (5.9)	2 (5.4)	3 (5.3)	3 (11.1)	0.874
Exposure keratopathy	2^a^ (8.3)	0^ab^ (0.0)	0^ab^ (0.0)	0^b^ (0.0)	0^ab^ (0.0)	**0.030**
Diabetes	2 (8.3)	2 (11.8)	1 (2.7)	4 (7)	4 (14.8)	0.408
Autoimmune disease	0 (0.0)	0 (0.0)	2 (5.4)	2 (3.5)	0 (0.0)	0.805

Significant* p*- values are indicated in bold

The superscripted letters a, b, and c are used to indicate whether there were significant differences between groups in a single row. A common letter between groups indicates no significant difference, while the lack of a common letter indicates a significant difference

*IQR* Interquartile range

*SD* Standard deviation

Abbreviations: *GPBK* Gram-positive bacterial keratitis; *GNBK* Gram-negative bacterial keratitis; *CDBK* Clinically diagnosed bacterial keratitis; *FK* Fungal keratitis; and *MFBK* Mixed fugal and bacterial keratitis; *LASIK* Laser in-situ keratomileusis; *TPK* Therapeutic penetrating keratoplasty

### Microbiological analysis

**Fig. 2:**
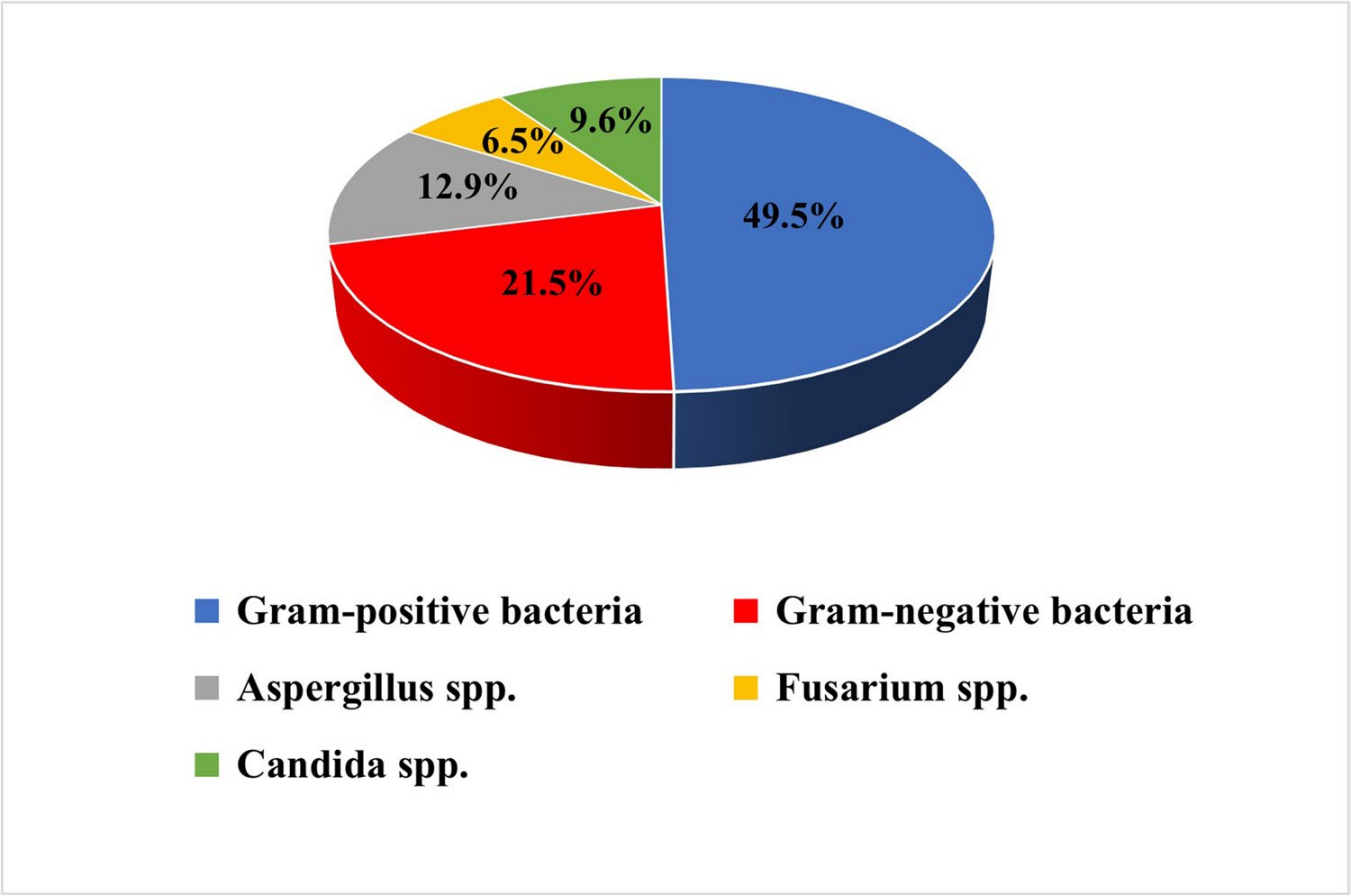
The prevalence of isolated microorganisms in microbial keratitis

The culture positivity rate reached 60% (93/155). Bacteria were the most frequent causative agents (71%), with a detected predominance of GP bacteria (49.5%), while 21.5% of the isolates were GN bacteria. The CoNS were the most common bacteria (30.1%), followed by PA (14%). Fungi were the second most encountered agents (29%). Filamentous fungi were more prevalent (19.4%) than *Candida* spp. (9.6%), with *Aspergillus* spp. (12.9%) being more predominant than *Fusarium* spp. (6.5%) (Fig. 2). Mixed growth of microorganisms represented 10.8% (10/93) and was distributed as follows: bacteria with fungi (7/10), and *Acanthamoeba* with bacteria (3/10).

**Fig. 3:**
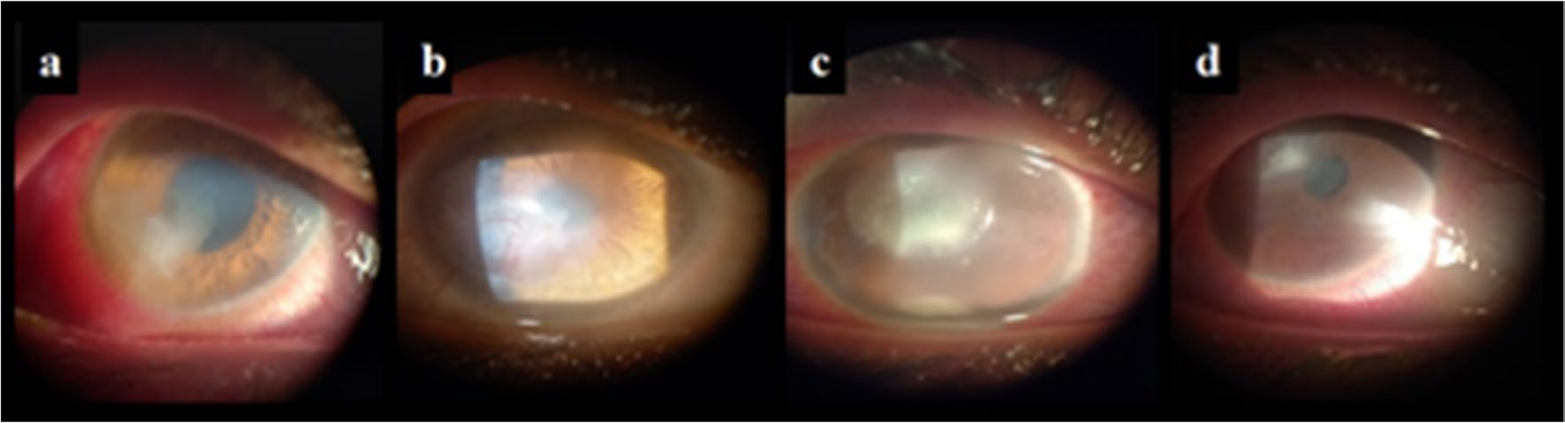
Slit-lamp biomicroscope photographs of culture-positive bacterial keratitis patients (**a-d**). **a**, **b** CoNS keratitis case, **a** before treatment, a paracentral ulcer (3.5 mm) with infiltration is shown. **b** After 32 days on levofloxacin, the ulcer healed with vascularized opacity. **c**, **d** MDR*-*PA (P4) keratitis case, **c** before treatment, a mean ulcer size and infiltrate of 3.25 mm, and hypopyon are shown. **d** After 30 days on GEN and ciprofloxacin, the epithelial defect healed with little infiltration remaining and opacity

The cumulative antibiogram of isolated GP bacteria from keratitis patients is represented in Table 2. Among the GP bacteria, CoNS were the most prevalent isolated bacteria (60.9%) (28/46), where MR-CoNS represented 25% of the identified bacteria. *Streptococci* spp. were the second most common (21.7%), followed by MRSA (8.7%). In general, GP bacteria were highly susceptible to VAN (93.3%) and linezolid (92.9%), but had lower susceptibilities to MOX (75%), and to other members of fluoroquinolones (31.6–57.7%), aminoglycosides (45.4–66.7%), and cephalosporins (17.1–20.7%). Methicillin-resistant strains (MR-CoNS and MRSA) represented 23.9% (11/46) of GP bacteria. The isolated CoNS (including MR-CoNS) were 100% susceptible to VAN and linezolid, highly susceptible to MOX (90.9%) but showed variable resistance to other members of fluoroquinolones (53.4–75%), aminoglycosides (42.3–60%), and cephalosporins (82.4–86.4%). All the CoNS isolates were resistant to fusidic acid (100%). The clinical course of a diagnosed 19-year-old male patient with CoNS keratitis presenting with an ulcer that did not improve on empiric MOX is described in Fig. 3a and b. Following C&S, MOX was switched into levofloxacin, where the post-treatment BCVA drastically improved from CF (0.014) to 0.5. Among the GP strains, *Streptococcus pneumonia* had the highest susceptibilities to VAN, linezolid, and teicoplanin (100% each). A percentage of 50% of MRSA were resistant to azithromycin and MOX.

**Table 2:** Cumulative antibiogram of Gram-positive bacteria isolated from patients with keratitis, their frequencies, and percentage susceptibilities to different antibiotics

		Percentage susceptibility % (*n*)^b^								
		β- lactams				Fluoroquinolones				
Gram-positive bacteria	*N*^a^	CAZ	CRO	CTX	OXA	CIP	GAT	LVX	MOX	OFX
MS-CoNS	21	20 (15)	30 (10)	30 (10)	100 (10)	54.5 (11)	20 (10)	54.5 (11)	88.9 (9)	66.7 (18)
MR-CoNS	7	0 (7)	0 (7)	0 (7)	0 (7)	25 (4)	50 (2)	50 (2)	100 (2)	33.3 (6)
Total CoNS	28	13.6 (22)	17.6 (17)	17.6 (17)	58.8 (17)	46.6 (15)	25 (12)	53.8 (13)	90.9 (11)	58.3 (24)
*S. pneumoniae*	8	50 (6)	60 (5)	40 (5)	50 (2)	66.7 (6)	50 (4)	83.3 (6)	60 (5)	71.4 (7)
*S. pyogenes*	1	‒	‒	‒	‒	‒	0	100	‒	0
*S. viridans*	1	‒	0	‒	‒	‒	‒	100	‒	100
Total *Streptococcus* spp.	10	50 (6)	50 (6)	40 (5)	50 (2)	66.7 (6)	40 (5)	87.5 (8)	60 (5)	66.7 (9)
MRSA	4	0 (4)	0 (4)	0 (4)	0 (4)	50 (2)	‒	33.3 (3)	50 (2)	25 (4)
RGM	3	0 (3)	0 (2)	0 (2)	0 (1)	100 (2)	50 (2)	0 (2)	50 (2)	50 (2)
*Corynebacterium* spp.	1	‒	‒	‒	‒	‒	‒	‒	‒	‒
Total GP bacteria	46	17.1 (35)	20.7 (29)	17.9 (28)	45.8 (24)	56 (25)	31.6 (19)	57.7 (26)	75 (20)	56.4 (39)

	Percentage susceptibility % (*n*)^b^									
	Aminoglycosides			Others						
Gram-positive bacteria	AMK	GEN	TOB	AZM	CH	TE	FA	LZD	TEC	VAN
MS-CoNS	100 (1)	65 (20)	40 (5)	30 (10)	‒	50 (2)	0 (4)	100 (2)	30 (10)	100 (21)
MR-CoNS	‒	33.3 (6)	‒	50 (6)	‒	50 (2)	0 (2)	100 (3)	80 (5)	100 (7)
Total CoNS	100 (1)	57.7 (26)	40 (5)	37.5 (16)	‒	50 (4)	0 (6)	100 (5)	46.7 (15)	100 (28)
*S. pneumoniae*	60 (5)	42.9 (7)	75 (4)	28.6 (7)	50 (4)	60 (5)	25 (4)	100 (3)	100 (2)	100 (8)
*S. pyogenes*	‒	‒	‒	100	100	‒	‒	‒	‒	100
*S. viridans*	‒	‒	‒	‒	‒	‒	‒	100	100	0
Total *Streptococcus* spp.	60 (5)	42.9 (7)	75 (4)	37.5 (8)	60 (5)	60 (5)	25 (4)	100 (4)	100 (3)	90 (10)
MRSA	100 (1)	25 (4)	0 (1)	50 (4)	0 (1)	66.7 (3)	0 (2)	100 (3)	100 (3)	100 (4)
RGM	0 (2)	50 (2)	0 (1)	66.7 (3)	0 (1)	0 (2)	0 (2)	0 (1)	0 (1)	0 (2)
*Corynebacterium* spp.	‒	‒	‒	‒	‒	‒	‒	100	‒	100
Total GP bacteria	66.7 (9)	51.3 (39)	45.4 (11)	41.9 (31)	57.1 (7)	50 (14)	7.1 (14)	92.9 (14)	59.1 (22)	93.3 (45)

^a^ number of identified isolates

^b^ number of isolates included for culture and sensitivity against antibiotics using the disc diffusion method; Isolates were tested against different antibiotics and the results were interpreted according to CLSI criteria and antibiotic disc availability

‒ No available data

Abbreviations: *MS-CoNS* Methicillin-sensitive coagulase-negative staphylococci; *MR-CoNS* Methicillin-resistant coagulase-negative staphylococci; *MRSA* Methicillin-resistant-*Staphylococcus aureus*; *RGM* Atypical rapidly growing mycobacteria; *AMK* Amikacin; *AZM* Azithromycin; *CAZ* Ceftazidime; *CRO* Ceftriaxone; *CH* Chloramphenicol; *CIP* Ciprofloxacin; *CTX* Cefotaxime; *FA* Fusidic acid; *GAT* Gatifloxacin; *GEN* Gentamicin; *LVX* Levofloxacin; *LZD* Linezolid; *MOX* Moxifloxacin; *OFX* Ofloxacin; *OXA* Oxacillin; *TE* Tetracycline; *TEC* Teicoplanin; *TOB* Tobramycin; and *VAN* Vancomycin

The cumulative antibiogram of isolated GN bacteria from MK patients is represented in Table 3.

**Table 3:** Cumulative antibiogram of Gram-negative bacteria isolated from patients with keratitis, their frequencies, and percentage susceptibilities to different antibiotics

		Percentage susceptibility % (*n*)^b^							
		β- lactams			Fluoroquinolones				
Gram-negative bacteria	*N*^a^	CAZ	CRO	CTX	CIP	GAT	LVX	MOX	OFX
*P. aeruginosa*	13	16.7 (12)	20 (5)	0 (4)	50 (10)	100 (8)	77.8 (9)	66.7 (6)	57.1 (7)
*E. coli*	6	0 (4)	0 (3)	0 (3)	60 (5)	66.7 (3)	66.7 (3)	0 (1)	100 (3)
*Proteus* spp*.*	1	0	‒	‒	‒	‒	0	‒	‒
Total GN bacteria	20	11.8 (17)	12.5 (8)	0 (7)	53.3 (15)	90.9 (11)	75 (12)	57.1 (7)	70 (10)

	Percentage susceptibility % (*n*)^b^					
	Aminoglycosides			Others		
Gram-negative bacteria	AMK	GEN	TOB	AZM	CH	TE
*P. aeruginosa*	100 (4)	63.6 (11)	55.6 (9)	0 (1)	16.7 (6)	0 (3)
*E. coli*	‒	16.7 (6)	100 (6)	100 (1)	33.3 (3)	50 (2)
*Proteus* spp*.*	‒	0	100	‒	‒	‒
Total GN bacteria	100 (4)	44.4 (18)	75 (16)	50 (2)	22.2 (9)	20 (5)

^a^ number of identified isolates

^b^ number of isolates included for culture and sensitivity against antibiotics using the disc-diffusion method; Isolates were tested against different antibiotics and the results were interpreted according to CLSI criteria and antibiotic disc availability

‒ No available data

Abbreviations: *AMK* Amikacin; *AZM* Azithromycin; *CAZ* Ceftazidime; *CRO* Ceftriaxone; *CH* Chloramphenicol; *CIP* Ciprofloxacin; *CTX* Cefotaxime; *GAT* Gatifloxacin; *GEN* Gentamicin; *LVX* Levofloxacin; *MOX* Moxifloxacin; *OFX* Ofloxacin; *TE* Tetracycline; and *TOB* Tobramycin

Among GN bacteria, PA isolates were the mostly recovered (65%) (13/20), followed by other members of *Enterobacteriaceae* (35%). Gram-negative bacteria were highly susceptible to gatifloxacin (90.9%) and amikacin (100%) but exhibited variable resistance to other members of fluoroquinolones (25%-46.7%), aminoglycosides (25%-55.6%), and cephalosporins (87.5–100%). All of the isolated PA were susceptible to gatifloxacin (100%) but lower susceptibilities to MOX (66.7%) and to third-generation fluoroquinolones (50–77.8%) were detected. They displayed, as well, higher resistance rates to CAZ (83.3%) compared to GEN (36.4%).

A percentage of 30.8% (4/13) of isolated PA were MDR-PA, while 50% (3/6) of *E. coli* were ESCR*-E. coli*. Antibiotic resistance patterns of MDR-PA, P1 to P4, and ESCR- *E. coli*, E1 to E3, isolated from 4 and 3 different keratitis patients, respectively, and their responses to empiric and culture-guided therapies are shown in supplementary Table S1. Drug therapy failed in 25% (1/4) of MDR-PA keratitis cases (P3), where the ulcer did not respond to empiric MOX and required TPK surgical intervention after corneal melting. The graft’s epithelial defect had eventually healed upon treatment with tobramycin following the results obtained from C&S. Meanwhile, drug therapy succeeded in all ESCR-*E. coli* keratitis cases.

The clinical course of a 17-year-old female infected with an MDR-PA (P4) keratitis with history of CL wear is shown in Fig. 3c and d. In this case, the ulcer improved on empiric GEN + VAN, GEN was continued while VAN was replaced by ciprofloxacin, according to C&S results. The BCVA drastically improved from HM (0.0052) to 0.05 post-treatment.

The cumulative antibiogram of isolated fungi from keratitis patients is shown in Table 4.

**Table 4:** Cumulative antibiogram of fungi isolated from patients with keratitis, their frequencies, and percentage susceptibilities to antifungals

		Percentage susceptibility % (*n*)^b^			
Fungal isolates	*N*^a^	amphotericin B	fluconazole	itraconazole	voriconazole
*Aspergillus* spp.	12	‒	0 (3)	‒	80 (5)
*Fusarium* spp.	6	0 (1)	50 (2)	100 (2)	100 (2)
Total filamentous fungi	18	0 (1)	20 (5)	100 (2)	85.7 (7)
*Candida* spp.	9	100 (3)	100 (3)	100 (3)	100 (3)
Total fungi	27	75 (4)	50 (8)	100 (5)	90 (10)

^a^ number of identified isolates

^b^ number of tested isolates for culture and sensitivity against antifungals using the disc-diffusion method. Isolates were tested against antifungals according to CLSI criteria and disc availability

‒ no available data

Filamentous fungi were highly susceptible to VRC (85.7%), with *Aspergillus* spp. having a susceptibility of 80%. The isolated *Candida* spp. showed 100% susceptibility to all tested antifungals. The isolated fungi showed 100% susceptibility to itraconazole and 90% to voriconazole, while least percentage of susceptibility was displayed by fluconazole (50%).

Bacterial keratitis, FK, and MFBK patients treated with the studied empiric antimicrobial regimens (*n* = 123), were compared for baseline characteristics and clinical picture (supplementary Tables S2-S4) and for treatment outcomes (supplementary Tables S5-S7). There were no statistically significant differences between empiric groups at baseline characteristics and clinical picture (*p* > 0.05) except in BK, where the CAZ + VAN-treated group (4.61 ± 2.03) had larger size of ulcers (in mm) as compared with MOX group (2.72 ± 1.44) (*p* = 0.012). However, ulcer sizes in the GEN + VAN- (4.13 ± 2.31) and CAZ + VAN-treated groups were comparable (supplementary Table S2).

### Clinical response, primary and secondary outcomes

**Fig. 4:**
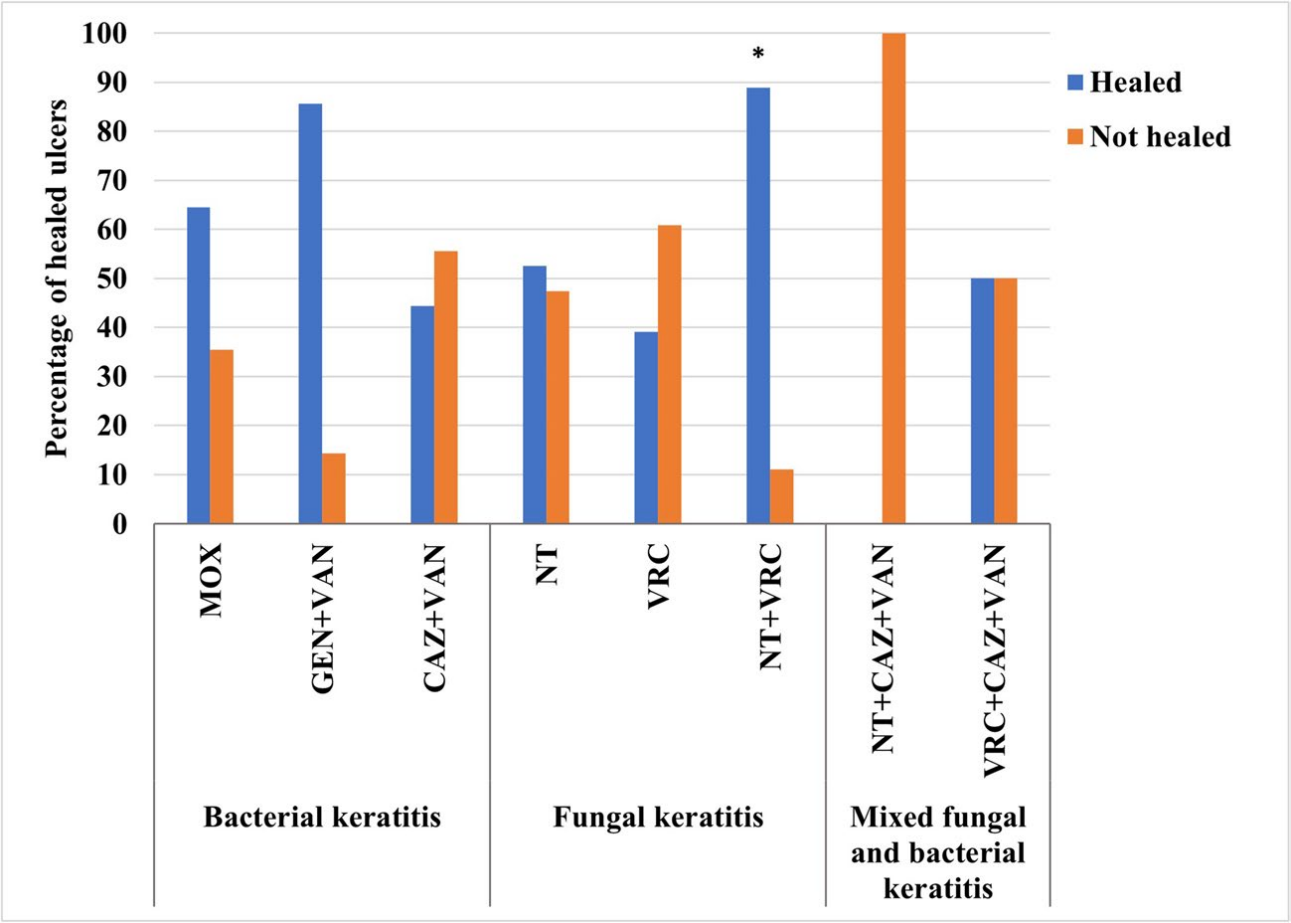
Percentage of corneal ulcers healed in microbial keratitis patients treated with antimicrobial regimens. Antimicrobial regimens were MOX: moxifloxacin 0.5%, GEN + VAN: fortified gentamicin 1.4% + vancomycin 5%, CAZ + VAN: fortified ceftazidime 5% + vancomycin 5%, NT: natamycin 5%, VRC: voriconazole 1%, NT + VRC: natamycin 5% + voriconazole 1%, NT + CAZ + VAN: natamycin 5% + ceftazidime 5% + vancomycin 5%, and VRC + CAZ + VAN: voriconazole 1% + ceftazidime 5% + vancomycin 5%. The p-value indicates significance at *p < 0.05 among treatment groups

The 49 BK patients were either treated with empiric MOX (*n* = 31), GEN + VAN (*n* = 9), or CAZ + VAN (*n* = 9) and resulted in the healing of 63.8% of their ulcers (*n* = 30). Most of the ulcers treated with GEN + VAN improved (8/9) (88.9%), with 2 of the improved cases had their regimen with VAN interrupted and switched into GEN plus ciprofloxacin after the C&S results revealed the presence of PA*.* Therefore, the GEN + VAN-treated group had a final percentage of healed ulcers of 6/7 (85.7%), which was non-significantly higher than the groups treated with CAZ + VAN (4/9) (44.4%) or MOX (20/31) (64.5%) (*p* = 0.259) (Fig. 4).

**Fig. 5:**
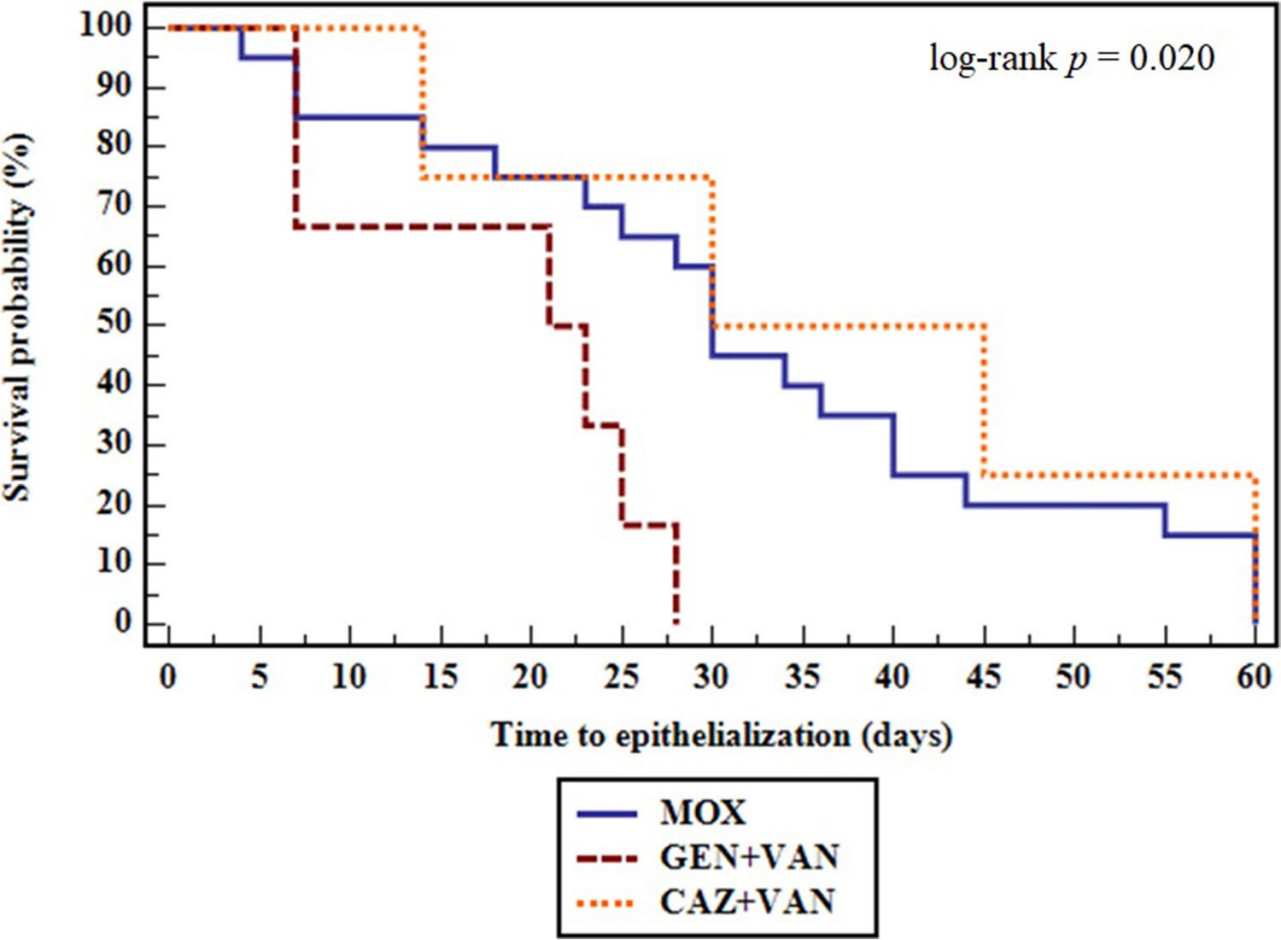
Kaplan–Meier survival curve in bacterial keratitis patients comparing time-to-epithelialization between antibiotic treatment groups. Antibiotic treatment groups were MOX: moxifloxacin 0.5%, GEN + VAN: fortified gentamicin 1.4% + vancomycin 5%, and CAZ + VAN: fortified ceftazidime 5% + vancomycin 5%

In the survival analysis, Kaplan–Meier curve shows that the GEN + VAN-treated group had the fastest ulcer healing time, with 50% of treated patients with GEN + VAN having their ulcers healed at a significantly shorter median time-to-epithelialization, 21 days, as compared to the CAZ + VAN- or MOX-treated groups (30 days each) (log- rank *p* = 0.020) (Fig. 5).

Frequency of corneal complications including opacities was comparable between antibiotic groups with non-significantly higher rates of corneal perforations, melting, and thinning/descemetocele (22.2% each) in the CAZ + VAN-treated group, but were absent in the GEN + VAN-treated group (*p* > 0.05) (supplementary Table S5).

**Fig. 6:**
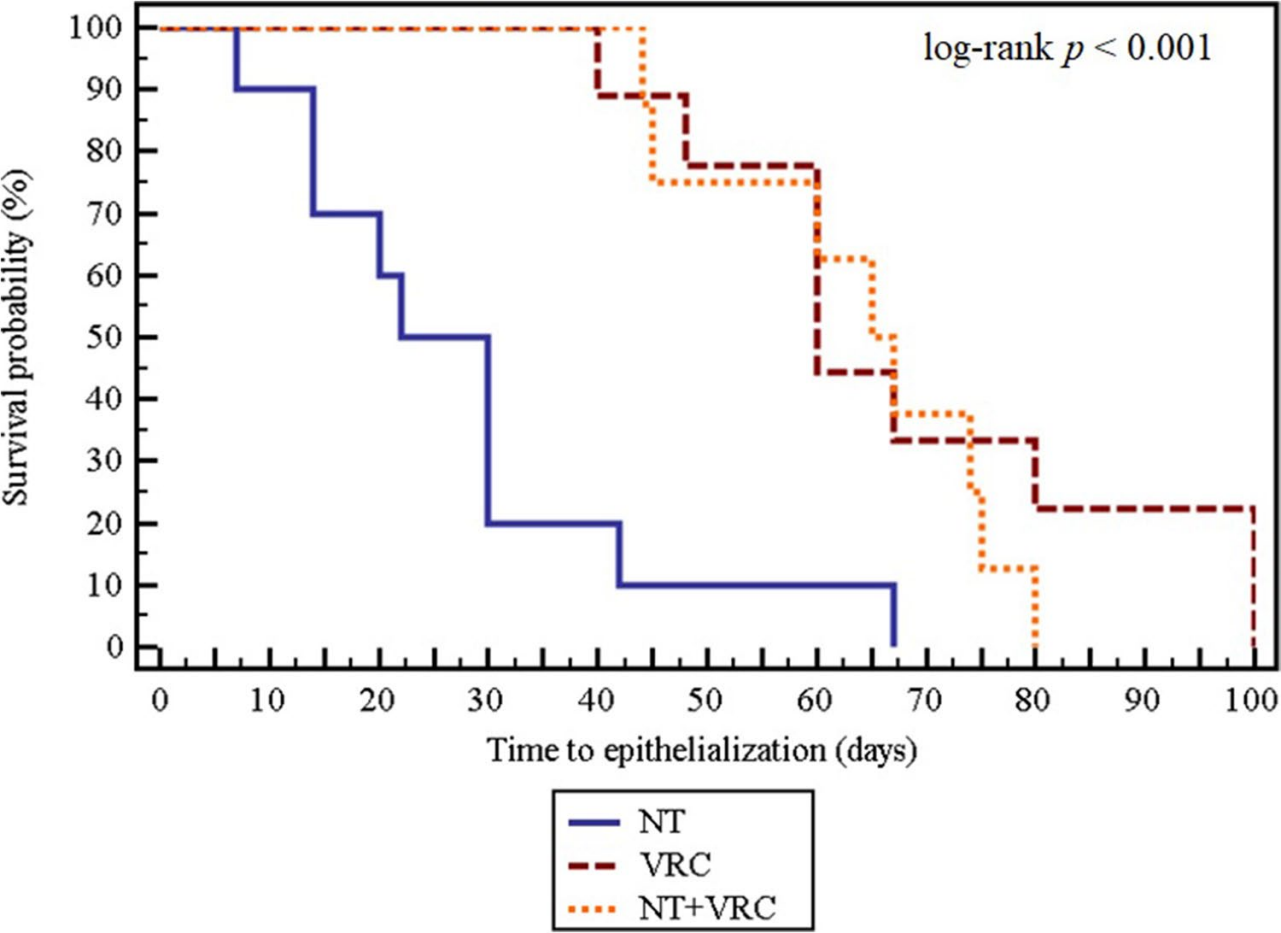
Kaplan–Meier survival curve in fungal keratitis patients comparing time-to-epithelialization between antifungal treatment groups. Antifungal treatment groups were NT: natamycin 5%, VRC: voriconazole 1%, and NT + VRC: natamycin 5% + voriconazole 1%

The 51 FK patients were either treated with empiric NT (*n* = 19), VRC (*n* = 23), or NT + VRC (n = 9) and resulted in 52.9% (27/51) of their ulcers healed. There was a statistically significant difference in percentages of healed ulcers in patients treated with NT + VRC, NT, and VRC (*p* = 0.036). Ulcer healing rate in the NT + VRC-treated group was significantly higher (88.9%) (8/9) when compared to the VRC-treated group (39.1%) (9/23) (*p* = 0.018), but non-significantly higher than the NT-treated group (52.6%) (10/19) (*p* = 0.098). The NT- and VRC-treated groups had comparable ulcer healing rates (*p* = 0.382) (Fig. 4). In the survival analysis, Kaplan–Meier curve shows that the NT-treated group had the fastest ulcer healing, with 50% of patients treated with NT having their ulcers healed at a significantly shorter median time-to-epithelialization, 22 days, as compared to 60 days on VRC and 65 days in the NT + VRC-treated groups (log-rank *p* < 0.001) (Fig. 6).

Frequency of corneal complications including opacities was comparable between antifungal groups, with non-significantly higher corneal perforations, melting, and thinning/descemetocele events in VRC-treated group (*p* > 0.05) (supplementary Table S6).

The 23 MFBK patients were either treated with empiric NT + CAZ + VAN (*n* = 7) or VRC + CAZ + VAN (*n* = 16) and resulted in only 8/23 (34.8%) of their ulcers healed, where interventions (either changing treatment or TPK) were initiated in 65.2% of keratitis cases. All the healed ulcers belonged to the VRC + CAZ + VAN-treated group (8/16) (50.0%) as compared with NT + CAZ + VAN-treated group (0.0%) (*p* = 0.052) (Fig. 4). Frequency of complications was comparable between groups (supplementary Table S7).

**Fig. 7:**
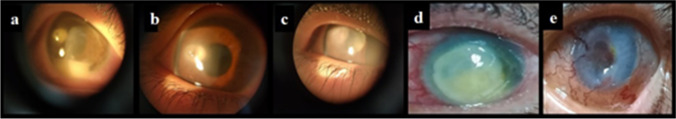
Slit-lamp biomicroscope photographs of a fungal keratitis case (**a-c**) and photographs of a mixed fungal and bacterial keratitis case (**d**, **e**). **a, b, c** Fungal keratitis case treated with natamycin 5% + voriconazole 1% (NT + VRC), **a** before treatment, a small paracentral epithelial defect (2 mm), with dense infiltrate, dense hypopyon and anterior chamber membrane are shown. **b** After 11 days of treatment, the ulcer improved, with decreased infiltration and resolved hypopyon, but it was complicated with descemetocele. **c** After 60 days, the ulcer healed with opacity and cataract. **d, e** Mixed fungal and bacterial keratitis case treated with VRC + CAZ + VAN, **d** before treatment, a moderate size ulcer (3.5 mm), with a larger size infiltrate is shown. **e** After 90 days of treatment, the ulcer epithelialized, but it was complicated with corneal melting

The clinical course of an 8-year-old female child, with a history of plant trauma, treated with NT + VRC, after being clinically diagnosed with FK, with the post-treatment BCVA remaining at HM (0.0052), is described in Fig. 7a, b, and c. The clinical course of a 65- year-old diabetic female, after being clinically diagnosed with MFBK, and treated with VRC + CAZ + VAN, with a post-treatment BCVA remaining at PL (0.0016), is shown in Fig. 7d and e.

In all groups, non-healed ulcers received interventions. In BK, most ulcers treated with CAZ + VAN did not respond and required interventions (55.6%). None of the treated patients with GEN + VAN had undergone TPK as compared to the CAZ + VAN- (33.3%) and MOX-treated groups (9.7%) (*p* = 0.136).

In FK patients, there was a statistically significant difference in the percentages of interventions received by patients treated with NT (47.4%), VRC (60.9%), or NT + VRC (11.1%) (*p* = 0.034), with significantly more interventions being initiated in the VRC-treated group, including TPK (30.4%), AC wash, IC/IS injections with antifungals (17.4%), change of treatment (21.7%), or cyanoacrylate corneal gluing (4.3%) as compared to the NT + VRC-treated group where the rates of the abovementioned interventions reached 11.1%, 11.1%, 0%, and 0%, respectively (*p* = 0.018).

**Fig. 8:**
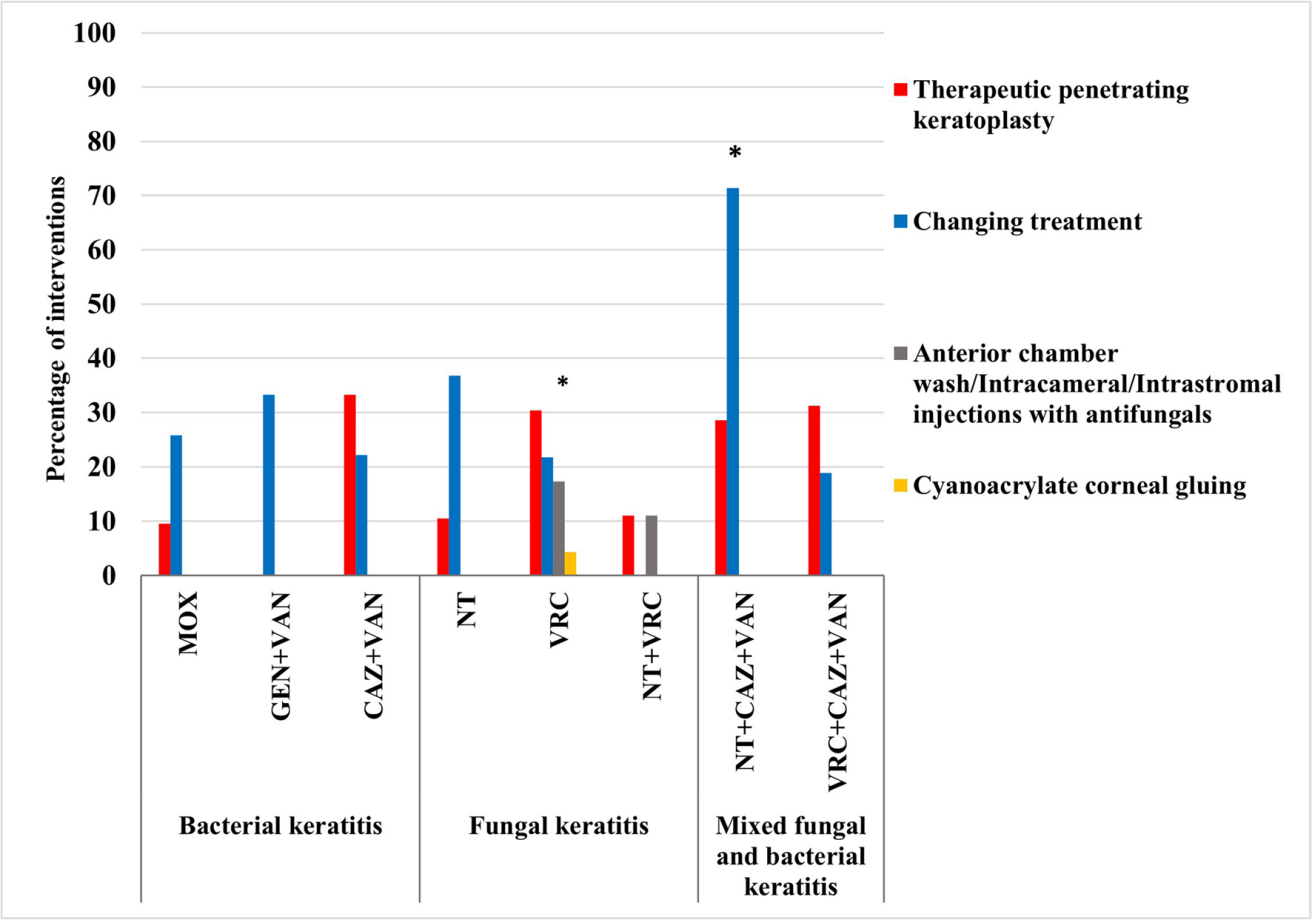
Types and frequencies of initiated interventions in non-healed ulcers in microbial keratitis patients treated with antimicrobial regimens. Antimicrobial regimens were MOX: moxifloxacin 0.5%, GEN + VAN: fortified gentamicin 1.4% + vancomycin 5%, CAZ + VAN: fortified ceftazidime 5% + vancomycin 5%, NT: natamycin 5%, VRC: voriconazole 1%, NT + VRC: natamycin 5% + voriconazole 1%, NT + CAZ + VAN: natamycin 5% + ceftazidime 5% + vancomycin 5%, and VRC + CAZ + VAN: voriconazole 1% + ceftazidime 5% + vancomycin 5%. The *p*-value indicates significance at *p < 0.05 among treatment groups

In MFBK, 65.2% of cases (*n* = 15) had undergone either a change of treatment (34.8%) or TPK (30.4%). All ulcers (100%) on NT + CAZ + VAN did not improve (*p* = 0.048), therefore received interventions compared with ulcers on VRC + CAZ + VAN (50%) (*p* = 0.052). Significantly more patients on NT + CAZ + VAN had their regimen changed (71.4%) compared with those on VRC + CAZ + VAN (18.75%) (*p* = 0.026) (Fig. 8).

**Fig. 9:**
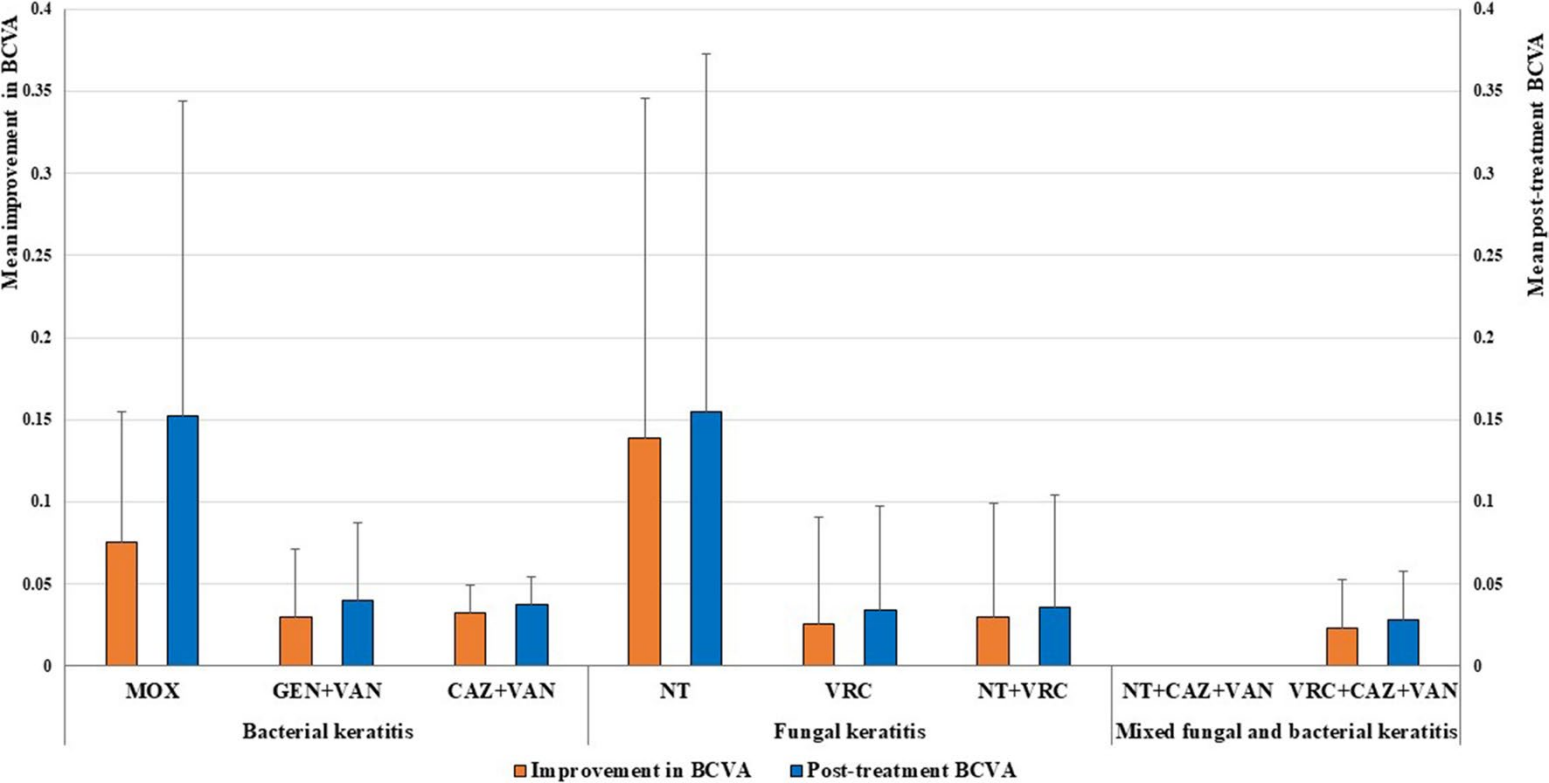
Means of improvement in the best-corrected visual acuity (BCVA) and the post-treatment BCVA in microbial keratitis patients treated with antimicrobial regimens. Antimicrobial regimens were MOX: moxifloxacin 0.5%, GEN + VAN: fortified gentamicin 1.4% + vancomycin 5%, CAZ + VAN: fortified ceftazidime 5% + vancomycin 5%, NT: natamycin 5%, VRC: voriconazole 1%, NT + VRC: natamycin 5% + voriconazole 1%, NT + CAZ + VAN: natamycin 5% + ceftazidime 5% + vancomycin 5%, and VRC + CAZ + VAN: voriconazole 1% + ceftazidime 5% + vancomycin 5%. All patients on NT + CAZ + VAN had their treatment changed, therefore values of BCVA were not available for this treated group. Error bars represent standard deviations

The means of BCVA improvement and post-treatment BCVA were non-significantly higher in the MOX-treated group when compared to the GEN + VAN- and CAZ + VAN-treated groups in BK and were non-significantly higher in the NT-treated group when compared to the VRC-, and NT + VRC- treated groups in FK (*p*-values > 0.05) (Fig. 9).

## Discussion

To the best of our knowledge, our study is the first to investigate antimicrobial resistance in MK in Alexandria, Egypt. Nevertheless, other studies explored antimicrobial resistance encountered in MK in different regions in the Middle East [18, 19]. In the current study, the clinical outcomes of GEN + VAN versus CAZ + VAN or MOX in BK, as well as the clinical outcomes of NT + VRC versus NT or VRC in FK, and those of NT + CAZ + VAN versus VRC + CAZ + VAN in MFBK, have been compared.

Patients on GEN + VAN had higher ulcer healing rates compared to MOX or CAZ + VAN, with more complications occurring in the latter group. Equivalent visual outcomes were found among all groups. Although differences in healing rates and complications were not statistically significant, due to the small sample size examined, they could have an important clinical impact. Our findings came in accordance with two larger studies conducted by Sharma et al. [13, 20], and Shah et al. [15] reporting the equivalence of topical fluoroquinolones monotherapy to fortified antibiotics combination in ulcer healing and BCVA. In the current study, GEN + VAN group had a significantly shorter median time-to-epithelialization, 21 days, as compared to MOX group, 30 days, or to CAZ + VAN group, 30 days. In contrast, Shah et al. reported indifference in healing times between groups. However, the time-to-epithelialization detected in our study agreed with the authors’ reported ulcer healing time in groups treated with cefazolin + tobramycin (24.5 days), gatifloxacin (27.5 days), or MOX (24 days) [15], and with the detected median time-to-epithelialization in BK detected by Moe et al. (25 days) [21]. In consistency with our findings regarding the effectiveness of GEN + VAN, fortified GEN 1.4% and cefazolin 5% combination was reported to be successful in non-responders to MOX 0.5% [22]. In contrast to studies reporting the increased ocular cytotoxicity induced by GEN [23], we observed fast epithelialization in keratitis patients treated with GEN + VAN. Our result was supported by the findings of Tsai et al. who observed that the preservative-free fortified GEN 1.4% had less cytotoxic effects on corneal epithelium and maintained good cell viability, when compared with MOX and other fluoroquinolone-containing ophthalmic solutions [24]. Accordingly, we recommend the empirical use of fortified GEN + VAN over CAZ + VAN in severe BK, based on the faster healing effect exerted by this combination and the higher detected GN bacterial resistance to CAZ as compared to GEN. This was reflected as poor clinical outcomes leading to lower healing rates and higher complications in the CAZ + VAN group when compared to the GEN + VAN group. The increased resistance to cephalosporins in ocular GN bacteria was previously reported [25, 26] and could be attributed to the low cost of these antibiotics, simple access, and their frequent prescription in Egyptian healthcare establishments [27].

Due to the high prevalence of methicillin-resistant GP bacteria in Egypt [28], VAN is routinely prescribed in combination as fortified eye drops in the cornea clinic, rather than cefazolin, hence unlike previous trials which evaluated fortified antibiotic combinations containing cefazolin [15, 20, 21], we rather studied the combined effect of fortified VAN. In line with previous studies reporting the prevalence of methicillin-resistant GP keratitis [26, 29], we detected 23.9% of methicillin resistance among isolated GP bacteria, and in accordance with prior findings in Europe, [30–32], CoNS was the main causative agent in BK in the current study. This resemblance in our findings could be ascribed to similarities in the climate, the urbanized communities, and risk factors [1].

We observed similar results to previous studies conducted in Alexandria and North India reporting that PA was the second most common bacteria isolated in MK and the most prevalent GN causative agent [33, 34]. Nevertheless, a study conducted in Nottingham, UK, during 2015 to 2019, reported that PA was the most common isolated bacteria, followed by *Staphylococci* spp. [35]. This difference could be attributed to the authors’ findings of CL wear as the main risk factor, hence the prevalence of PA. However, our study showed that non-CL related BK accounted for the highest percentage of culture-positive BK (66%), explaining the predominance of *Staphylococcus* spp. [35]. MDR-PA keratitis represents a treatment challenge as it leads to poor treatment outcomes [26]. In line with previous studies [26, 36], the isolated PA in the current study exhibited multi-drug resistance (MDR-PA). The development of MDR keratitis could be attributed to the widespread use of the broad-spectrum antibiotics in the prophylaxis and treatment of MK [26]. In our developed antibiogram, susceptibilities of isolated PA to MOX (66.7%), tobramycin (55.6%), and CAZ (16.7%) were supported by the susceptibilities reported by Khurana et al. reaching 50%, 61%, and 21% to the mentioned antibiotics, respectively [37]. Gram-negative bacteria exhibited greater susceptibility to gatifloxacin (90.9%) than to MOX (57.1%), where the isolated PA showed 100% susceptibility to gatifloxacin, a finding which is supported by the Antibiotic Resistance Monitoring in Ocular Microorganisms (ARMOR) study conducted in the USA (94.3%) [38], and with previous studies reporting that gatifloxacin has twice the in vitro activity against ocular PA as compared to MOX [39, 40]. Accordingly, we recommend gatifloxacin as first line empiric therapy over MOX in suspected GNBK or PA keratitis.

In line with previous reports of resistance trends among ocular GP bacterial isolates to MOX [38, 41], we detected moderate GP bacterial susceptibilities to MOX (75%), ciprofloxacin (56%) and levofloxacin (57.7%), a finding that was lower than the GP susceptibility rate to fluoroquinolones reported from England (79.2%) [25]. The discrepancy of antibiotics resistance rates in different locations could be attributed to differences in antibiotic prescribing patterns, genomic variations, and environmental factors [42]. We detected a resistance rate of MS-CoNS to MOX, in line with that reported by the ARMOR study (8.1%) [38]. We also noted a high GP susceptibility percentage to VAN (93.3%), similar to the previously reported study by Moledina et al. [25]. Since the in vitro susceptibilities could predict the clinical outcomes [4, 43], accordingly, the empiric use of third-generation fluoroquinolones in GPBK is not advocated and should be replaced by the administration of empiric MOX. In addition, empiric treatment with fortified VAN is recommended in clinically suspected methicillin-resistant keratitis.

In the obtained culture-positive keratitis, fungi were less prevalent than bacteria, in contrast to the study reported by Mabrouk et al. where fungi were the predominant microorganisms isolated in the Upper Egypt governorate of Minia. This could be attributed to the different geographical location, hotter climate, and rural nature of Minia, where fungi prevail, compared to the moderate climate and the urban nature of Alexandria [2]. However, we obtained a similar prevalence rate of *Aspergillus* spp. among fungi [2]. A trend of elevated fungi resistance to VRC has been previously documented [44, 45]. Similarly, we detected the in vitro fungi resistance to VRC reaching 10%. This could be explained by the increased resistance of environmental fungi to azole antifungals [45]. Resistance to antifungals is a clinical concern as it is associated with an increased incidence of treatment failure [45]. In FK, a higher number of ulcers healed significantly when topical VRC was combined with NT as compared to VRC monotherapy, a result consistent with that of Sharma et al. who concluded that adding topical VRC to NT was beneficial in non-responders to NT [46]. It was in accordance, as well, with the Mycotic Ulcer Treatment Trial I (MUTTI) which stated that VRC should not be used solely in *Fusarium* keratitis [14]. Consistent with Prajna et al. findings, we obtained comparable visual outcomes and corneal perforation among the three antifungal groups. Nevertheless, our study detected significantly shorter median time-to-epithelialization, 22 days, when administrating NT, as compared to 60 days in the VRC group, and 65 days in the NT + VRC group. In accordance with our results, the MUTTI subgroup analysis reported a significantly shorter healing time in the NT group compared to the VRC group in *Fusarium* spp. cases [14]. In addition, the median time-to-epithelialization detected in our study for the NT group closely aligns with that reported by Moe et al. (30 days) [21]. Our findings of indifference between NT and VRC and the superiority of NT + VRC combination could be explained by the findings of Sradhanjali et al. stating that VRC and NT have synergistic in vitro activity against all tested fungal species, implying that the administration of these antifungals in combination may have more clinical effectiveness than the use of single agents [47]. Thus, we advocate the use of empiric topical VRC and NT combination, rather than a single antifungal, especially in severe FK.

Associations between MK and risk factors among the different keratitis groups noticed in the current study could be related to those previously reported in the literature. The detected risk factors included trauma in MK [1], vegetative trauma, agricultural occupation, and male gender in FK [48], corneal foreign body in BK [49], female gender and CL wear in GNBK [48], and exposure keratopathy in BK [50].

In the current study, 65.2% of MFBK cases had undergone either a change of treatment (34.8%) or TPK (30.4%). Similarly, Ahn et al. confirmed that 52% of mixed keratitis cases demonstrated initial treatment failure, with 15% having undergone TPK [51].

Future studies are needed to confirm our findings and to investigate other unstudied regimens such as NT + VRC + CAZ + VAN or GEN + VAN in combination with antifungals. We acknowledge and understand the limitations of this study. These include the small number of tested isolates (< 30) for antimicrobial susceptibility in antibiograms and the cautious interpretation of the susceptibility results. Furthermore, we were not able to investigate the in vitro fungi susceptibility to NT due to the unavailability of NT antibiotic discs. Another limitation is that the empiric antibiotic regimens used in our study may be different to those utilized in other parts of the world. For instance, fortified cefazolin/tobramycin combination is considered the standard therapy for the management of severe BK in many countries [7, 20]. In addition, the study is not multi-centered, hence the microbiological spectrum and susceptibility results may not be extrapolated outside Alexandria.

Further studies might be also performed by conducting panfungal PCR and broad range bacterial PCR, as PCR represents an advanced method for the identification of fungal and bacterial species and for the detection of antimicrobial resistance. There are other factors that may influence the time-to-epithelialization of corneal ulcers other than the bacterial and fungal susceptibility to antibiotics. The vehicle type in which the antibiotics are dissolved, and fortified antibiotic-induced epithelial toxicity may delay corneal ulcer healing [23]. Therefore, antibiograms may be a better indicator for defining antimicrobial susceptibilities than time-to-epithelialization in MK [6].

## Conclusions

In conclusion, CoNS, PA, and *Aspergillus* spp. were found to be the most encountered microorganisms in MK in Alexandria. GP bacteria (including CoNS) showed high susceptibility to VAN, linezolid, and MOX. Hence, as first line, we recommend empiric topical MOX in suspected GP bacterial keratitis, and fortified VAN or linezolid in clinically suspected methicillin-resistant keratitis. GN bacteria (including PA) displayed high susceptibility to gatifloxacin, hence we recommend gatifloxacin as first line empiric therapy in CL wearers cases or cases with clinically suspected GN keratitis. Based on the low detected bacterial resistance to GEN as compared to CAZ, and the significantly faster ulcer healing observed in BK patients treated with GEN + VAN as compared to CAZ + VAN, we suggest empiric fortified GEN over CAZ, in combination with fortified VAN, in severe MK. Fungal keratitis patients on topical VRC + NT showed significantly better clinical outcomes with higher ulcer healing rates and fewer interventions compared with VRC. Thus, treatment with VRC and NT combination is advocated over single agents in severe fungal cases. In non-responsive ulcers to NT, we recommend the addition of VRC to NT rather than switching over to VRC.

Performing cultures of corneal scrapings in severe ulcers is essential for detecting the causative agents and to combat antimicrobial resistance through reducing the use of broad-spectrum empiric therapies. The prudent and judicious use of systemic and topical antibiotics cannot be overemphasized to decrease multidrug resistance. The regular development of antibiograms for monitoring bacterial resistance trends and for guiding the choice of appropriate empiric therapies should be encouraged. It is important to highlight that our recommendations of the most effective empiric therapies for microbial keratitis could potentially change in the future, subject to updates in bacterial and fungal resistance data, together with patients’ clinical responses to antimicrobials.

### Supplementary Information

Below is the link to the electronic supplementary material.

Supplementary file1 (DOCX 1.46 mb)

## Data Availability

All data generated or analyzed during this study are included in this published article/as supplementary information files. References [52–56] are cited in Supplementary File.

## Declarations

### Medical writing, editorial, and other assistance

We received no medical writing or editorial assistance.

### Thanking patient participants

We thank the study participants for their involvement in the study.

### Prior presentation

Authors confirm that there was no prior presentation of any material from the manuscript at conferences.
