# Supplementary material for: A comparison of antimicrobial regimen outcomes and antibiogram development in microbial keratitis: a prospective cohort study in Alexandria, Egypt
Source: Graefes Arch Clin Exp Ophthalmol. 2024 Jan 19;262(6):1865–82. doi: 10.1007/s00417-023-06362-0 (PMC11106157; doi:10.1007/s00417-023-06362-0)
Supplement: Supplementary file 1 — Supplementary file1 (DOCX 1.46 mb) [file 417_2023_6362_MOESM1_ESM.docx]

**A comparison of antimicrobial regimen outcomes and antibiogram development in microbial keratitis: A prospective cohort study in Alexandria, Egypt.**

Amira A. Nayel^1,2^, Noha A. Hamdy^1*^, Tamer H. Massoud^3^, and Nelly M. Mohamed^4^

**^1^** Department of Clinical Pharmacy and Pharmacy Practice, Faculty of Pharmacy, Alexandria University, Alexandria, Egypt

**^2^** Clinical Pharmacy Department, Alexandria Ophthalmology Hospital, Ministry of Health and Population of Egypt, Alexandria, Egypt

**^3^** Department of Ophthalmology, Faculty of Medicine, Alexandria University, Alexandria, Egypt

**^4^** Department of Microbiology and Immunology, Faculty of Pharmacy, Alexandria University, Alexandria, Egypt

*Corresponding author. Mailing address: Noha A. Hamdy, Department of Clinical Pharmacy and Pharmacy Practice, Faculty of Pharmacy, El-Khartoom Square, Azarita, Alexandria, Egypt

Phone: (+203) 4868482. Fax: (+203) 4871668.

e-mail: [noha.alaaeldine@alexu.edu.eg](mailto:noha.alaaeldine@alexu.edu.eg)

**emails & ORCID:**

Nelly M. Mohamed: [nelly.mohamed@alexu.edu.eg](mailto:nelly.mohamed@alexu.edu.eg), 0000-0001-9073-5032

Tamer H. Massoud: [tamer.massoud@alexmed.edu.eg](mailto:tamer.massoud@alexmed.edu.eg), 0000-0002-6660-1664

Noha A. Hamdy: [noha.alaaeldine@alexu.edu.eg](mailto:noha.alaaeldine@alexu.edu.eg), 0000-0002-4606-8567

Amira A. Nayel: [gs-amira.nayel@alexu.edu.eg](mailto:gs-amira.nayel@alexu.edu.eg), 0000-0002-7356-9287

**Supplementary Methods**

**1 Preparation and administration of fortified eye drops**

**2 Cultivation of corneal scrapings and identification of causative organisms**

**3 Antimicrobial susceptibility testing (AST)**

**Supplementary Tables and Figures**

**Table S1** Antibiotic resistance patterns of MDR-PA and ESCR- *E. coli* isolated from different keratitis patients, responses/intervention to empiric therapy, and the *in vitro* susceptibilities to antibiotics of proven clinical responsiveness

**Table S2** Baseline characteristics and clinical picture of empiric antibiotics regimens: MOX, fortified GEN +VAN, and fortified CAZ+VAN in patients with bacterial keratitis

**Table S3** Baseline characteristics and clinical picture of empiric antifungal regimens: NT, VRC, and NT+VRC in patients with fungal keratitis

**Table S4** Baseline characteristics and clinical picture of empiric antifungal and antibiotic regimens: NT+CAZ+VAN, and VRC+CAZ+VAN in patients with mixed fungal and bacterial keratitis

**Table S5** Treatment outcomes and responses to empiric antibiotics regimens: MOX, GEN +VAN, and CAZ+VAN in patients with bacterial keratitis

**Table S6** Treatment outcomes and responses to empiric antifungal regimens: NT, VRC, and NT+VRC in patients with fungal keratitis

**Table S7** Treatment outcomes and responses to empiric antifungal and antibiotic regimens: NT+CAZ+VAN and VRC+CAZ+VAN in patients with mixed fungal and bacterial keratitis

**Fig. S1** Cultivation of corneal scraping samples on different culture media

**Fig. S2** Microscopic identification of the causative microorganisms of microbial keratitis from cultured corneal scrapings under the microscope (oil lens magnification x 100)

**Fig. S3** Biochemical tests performed for the identification of isolated microorganisms from corneal scrapings of diagnosed patients with bacterial keratitis

**SUPPLEMENTARY METHODS**

**1 Preparation and administration of fortified eye drops**

Ceftazidime and gentamicin, which cover Gram-negative (GN) bacteria, were combined with vancomycin, which is used for covering Gram-positive (GP) bacteria, including methicillin-resistant MR *Staphylococci*. Fortified vancomycin and ceftazidime 50 mg/ml eye drops were compounded in the pharmacy by the reconstitution of each of the 500 mg vials into 10 mL of artificial tears. Fortified gentamicin 1.4% eye drops were prepared by adding 2 mL of gentamicin (80 mg/2ml solution for injection) into 4 mL artificial tears [52].

Topical voriconazole 1% eye drops, a broad- spectrum azole with a superior corneal penetration, was prepared by the reconstitution of Vfend^®^ 200 mg vial into 19 mL sterile water for injection (SWFI). All preparations were stored in the fridge at 2 to 8 ℃. Eye drops were administrated in a loading dose of an hourly application to achieve high corneal concentration. Upon improvement, the frequency of administration was reduced to every two hours. The application of different types of eye drops was spaced by 10 to 15 minutes [53].

**2 Cultivation of corneal scrapings and identification of causative organisms**

Following topical anesthesia, corneal scraping was e performed under the slit-lamp biomicroscope. Scrapings were extracted from the base and margins of ulcers, using a sterile Kimura spatula. Specimens were inoculated on brain-heart infusion (BHI) broth and selective agar culture media (blood, chocolate, MacConkey, and Sabouraud dextrose agar (SDA) for the growth of bacteria and fungi. All media were incubated aerobically at 37℃ for 7 days, except when using SDA which was incubated at 25℃ for 14 days, and chocolate agar which was incubated anaerobically in a candle jar containing 3-5% CO_2_. The cultivated media were examined daily for growth.

A culture was considered positive if growth of microorganisms was detected along the line of inoculation on at least one solid culture medium. Fungal hyphae were directly detected by examining the scraping sample in a 10% potassium hydroxide wet mount (**Fig. S1)**. The cultivation of microorganisms on culture media is shown in **Fig. S1**. For microscopic identification, bacterial colonies were Gram-stained, and fungal colonies were Giemsa-stained **(Fig. S2)**. Partially stained acid-fast bacilli were re-stained by Ziehl-Neelsen.

If GP bacteria were isolated, catalase and coagulase tests were performed, together with subculturing on Mannitol Salt agar (MSA) for isolate’s identification. GN bacteria were identified using biochemical tests: triple sugar iron agar, motility indole ornithine, lysine iron agar, urease, citrate, and oxidase tests **(Fig. S3)**.

**3 Antimicrobial susceptibility testing (AST)**

Antibiotic and antifungal susceptibility tests were performed by agar disc diffusion method (Kirby-Bauer) [54]in accordance with Clinical and Laboratory Standards Institute (CLSI) guidelines [55] **(Fig. S1)**. A 0.5 McFarland standard was utilized to standardize the inoculums’ turbidity. A sterile cotton swab was dipped into the suspension and used to inoculate sterile Muller Hinton agar plates. The antimicrobial discs were placed and incubated at 37°C for 18–24 h.

When testing bacterial isolates, the following antibiotic discs were used: cefoxitin 30 µg, ceftazidime 30 µg, ceftriaxone 30 µg, cefotaxime 30 µg, azithromycin 15 µg, ciprofloxacin 5 µg, levofloxacin 5 µg, ofloxacin 5 µg, moxifloxacin 5 µg, gatifloxacin 5 µg, amikacin 30 µg, gentamicin 10 µg, tobramycin 10 µg, chloramphenicol 30 µg, teicoplanin 30 µg, tetracycline 30 µg, vancomycin 30 µg, linezolid 30 µg, and fusidic acid 10 µg. Whereas the antifungal discs used were: fluconazole 25 µg, voriconazole 1 µg, itraconazole 10 µg, and amphotericin B 100 µg.

The inhibition zone diameter was measured by a caliper, and the *in vitro* susceptibilities were interpreted as sensitive/susceptible “S”, intermediate “I” and resistant “R” according to the CLSI breakpoints. Owing to a limited availability of discs, not all isolates were tested against all discs.

For *Staphylococcus* species, cefoxitin (30 µg) discs were added; staphylococci isolates that demonstrated resistance to cefoxitin were considered MR, either MR-CoNS or MRSA.

*Escherichia coli* isolates that recorded R or I to one of the following antibiotics: cefepime, ceftriaxone, cefotaxime, and ceftazidime were classified as extended-spectrum cephalosporin-resistant *E. coli* (ESCR-*E. coli*). *Pseudomonas aeruginosa* isolates that recorded R or I when tested against one of these antibiotic categories: extended-spectrum cephalosporin (cefepime, ceftazidime), fluoroquinolones (ciprofloxacin, levofloxacin), or aminoglycosides (amikacin, gentamicin, tobramycin) were classified as multidrug-resistant *Pseudomonas aeruginosa* (MDR-PA) [56].

**Supplementary Tables**

**Table S1** Antibiotic resistance patterns of MDR-PA and ESCR- *E. coli* isolated from different keratitis patients, responses/intervention to empiric therapy, and the *in vitro* susceptibilities to antibiotics of proven clinical responsiveness

| Isolate | Antibiotic resistance patterns | Empiric therapy | Response to empiric therapy  /Intervention | *In vitro* susceptible antibiotic of proven clinical responsiveness and healing outcome |
| --- | --- | --- | --- | --- |
| P1 | CAZ CRO CTX **CIP** GEN | MOX | Nonresponsive | TOB and GAT |
| P2 | **CAZ CIP** GEN **TOB** | MOX | Improved | MOX |
| P3 | CRO CIP LVX GEN | MOX | Cornea melted/TPK | TOB |
| P4 | CAZ **LVX** TOB | GEN+VAN | Improved | GEN and CIP |
| E1 | **CAZ CRO** | GAT | Improved | GAT |
| E2 | CAZ CRO | GEN+VAN | Improved | GEN and CIP |
| E3 | CAZ CRO CTX | CAZ+VAN | Nonresponsive | GEN and CIP |

The bold format indicates intermediate susceptibility

MDR-PA: multidrug-resistant *Pseudomonas aeruginosa*

ESCR- *E. coli:* extended-spectrum cephalosporin-resistant *E. coli*

P: multidrug-resistant *Pseudomonas aeruginosa* isolated from different keratitis patients

E: Extended-spectrum cephalosporin-resistant *E. coli* isolated from different keratitis patients

Abbreviations: CAZ: ceftazidime, CRO: ceftriaxone, CIP: ciprofloxacin, CTX: cefotaxime, GAT: gatifloxacin, GEN: gentamicin, LVX: levofloxacin, MOX: moxifloxacin, TOB: tobramycin

**Table S2** Baseline characteristics and clinical picture of empiric antibiotics regimens: MOX, fortified GEN +VAN, and fortified CAZ+VAN in patients with bacterial keratitis

| Parameters | MOX  (n = 31) N (%) | GEN+VAN  (n = 9) N (%) | CAZ+VAN  (n = 9) N (%) | *p-value* |
| --- | --- | --- | --- | --- |
| Sex  Male  Female | 20 (64.5)  11(35.5) | 4 (44.4)  5 (55.6) | 5 (55.6)  4 (44.4) | 0.604 |
| Age (years)  Median (IQR) | 44 (28 – 57) | 37(31 – 63) | 47(38 – 60) | 0.863 |
| Mean ± SD | 44.26 ± 18.87 | 45.0 ± 20.49 | 48.22 ± 19.42 |  |
| Onset (days) |  |  |  |  |
| median (IQR) | 9 (3.5 – 22) | 7 (4 – 10) | 15 (8 – 30) | 0.280 |
| Ulcer site |  |  |  |  |
| No ulcer | 2 (6.5) | 1 (11.1) | 0 (0.0) | 0.428 |
| Central | 15 (48.4) | 5 (55.6) | 8 (88.9) |  |
| Paracentral | 9 (29.0) | 3 (33.3) | 1 (11.1) |  |
| Peripheral | 5 (16.1) | 0 (0.0) | 0 (0.0) |  |
| Ulcer depth | (n=29) | (n=8) | (n=9) |  |
| Superficial 2/3 of stroma | 27 (93.1) | 8 (100.0) | 7 (77.8) | 0.300 |
| Posterior 1/3 of stroma | 2 (6.9) | 0.0 (0.0) | 2 (22.2) |  |
| Ulcer size (mm) | (n = 29) | (n = 8) | (n = 9) | **0.017** |
| Median (IQR) | 2.50 (1.50 – 3.50) | 4.25 (2.50 – 5.25) | 3.50 (3.50 – 5.50) | **0.012^*^** |
| Mean ±SD | 2.72 ^b^ ± 1.44 | 4.13 ^ab^ ± 2.31 | 4.61 ^a^ ± 2.03 | 0.680^#^ |
| Small (≤2mm) | 12 (41.4) | 2 (25.0) | 0 (0.0) | 0.076 |
| Medium (>2 to≤ 6) | 16 (55.2) | 5 (62.5) | 8 (88.9) |  |
| Large (>6 mm) | 1 (3.4) | 1 (12.5) | 1 (11.1) |  |
| Infiltrate size |  |  |  |  |
| ≤ ulcer size | 23 (74.2) | 7 (77.8) | 7 (77.8) | 1.000 |
| > ulcer size | 8 (25.8) | 2 (22.2) | 2 (22.2) |  |
| Hypopyon | 7 (22.6) | 6 (66.7) | 4 (44.4) | 0.051 |
| Abscess | 2 (6.5) | 0 (0.0) | 0 (0.0) | 1.000 |
| BCVA at baseline |  |  |  |  |
| median (IQR) | 0.01 (0.01 – 0.01) | 0.01 (0.01 – 0.01) | 0.01 (0.01 – 0.01) | 0.544 |
| mean ± SD | 0.055 ± 0.152 | 0.018 ± 0.032 | 0.016 ± 0.032 |  |
| Microbiological results |  |  |  |  |
| Gram-positive bacteria | 7 (22.6) | 2 (22.2) | 1 (11.1) | 0.884 |
| Gram-negative bacteria | 5 (16.1) | 2 (22.2) | 1 (11.1) | 0.875 |
| Clinically diagnosed bacterial keratitis | 19 (61.3) | 5 (55.6) | 7 (77.8) | 0.703 |

Significant *p-* values are in bold

Medians with different letters are significant

**p-*value comparing ulcer size between CAZ+VAN and MOX groups

# *p-*value comparing ulcer size between CAZ+VAN and GEN+VAN groups

Abbreviations: BCVA: best-corrected visual acuity, IQR: interquartile range, SD: standard deviation,

MOX: moxifloxacin 0.5%, fortified GEN+VAN: gentamicin 1.4% + vancomycin 5%, fortified CAZ+VAN: ceftazidime 5% + vancomycin 5%

**Table S3** Baseline characteristics and clinical picture of empiric antifungal regimens: NT, VRC and NT+VRC in patients with fungal keratitis

| Parameters | NT  (n = 19) N (%) | VRC  (n = 23) N (%) | NT+VRC  (n = 9) N (%) | *p-value* |
| --- | --- | --- | --- | --- |
| Sex  Male  Female | 15 (78.9)  4 (21.1) | 13 (56.5)  10 (43.5) | 7 (77.8)  2 (22.2) | 0.240 |
| Age (years)  Median (IQR) | 49 (39.5 – 60.5) | 57 (47 – 63.5) | 44 (40 – 47) | 0.158 |
| Mean ± SD | 49.32 ± 12.09 | 52.26 ± 16.70 | 40.78 ± 15.53 |  |
| Onset (days) |  |  |  |  |
| median (IQR) | 7 (3 – 15) | 10 (3 – 22) | 14 (10 – 30) | 0.511 |
| Ulcer site |  |  |  |  |
| No ulcer | 0 (0.0) | 1 (4.3) | 0 (0.0) | 0.257 |
| Central | 10 (52.6) | 16 (69.6) | 6 (66.7) |  |
| Paracentral | 9 (47.4) | 6 (26.1) | 2 (22.2) |  |
| Peripheral | 0 (0.0) | 0 (0.0) | 1 (11.1) |  |
| Ulcer depth | (n = 19) | (n = 22) | (n = 9) |  |
| Superficial 2/3 of stroma | 11 (57.9) | 12 (54.5) | 4 (44.4) | 0.866 |
| Posterior 1/3 of stroma | 8 (42.1) | 10 (45.5) | 5 (55.6) |  |
| Ulcer size (mm) | (n = 19) | (n = 22) | (n = 9) |  |
| Median (IQR) | 3.0 (2.25 – 5.0) | 3.50 (3.50 – 5.0) | 3.0 (3.0 – 5.0) | 0.169 |
| Mean ±SD | 3.66 ± 2.30 | 4.39 ± 1.82 | 3.61 ± 1.32 |  |
| Small (≤2mm) | 5 (26.3) | 1 (4.5) | 2 (22.2) | 0.246 |
| Medium (>2 to≤ 6) | 12 (63.2) | 17 (77.3) | 7 (77.8) |  |
| Large (>6 mm) | 2 (10.5) | 4 (18.2) | 0 (0.0) |  |
| Infiltrate size |  |  |  |  |
| ≤ ulcer size | 9 (47.4) | 7 (30.4) | 4 (44.4) | 0.502 |
| > ulcer size | 10 (52.6) | 16 (69.6) | 5 (55.6) |  |
| Hypopyon | 7 (36.8) | 12 (52.2) | 4 (44.4) | 0.625 |
| Abscess | 4 (21.1) | 4 (17.4) | 1 (11.1) | 1.000 |
| BCVA at baseline |  |  |  |  |
| median (IQR) | 0.01 (0.01 – 0.01) | 0.01 (0.01 – 0.01) | 0.01 (0.01 – 0.01) | 0.479 |
| mean ± SD | 0.016 ± 0.024 | 0.009 ± 0.010 | 0.006 ± 0.003 |  |

Abbreviations: BCVA: best-corrected visual acuity, IQR: interquartile range, SD: standard deviation,

NT: natamycin 5%, VRC: voriconazole 1%, NT+VRC: natamycin 5% + voriconazole 1%

**Table S4** Baseline characteristics and clinical picture of empiric antifungal and antibiotic regimens: NT+CAZ+VAN, and VRC+CAZ+VAN in patients with mixed fungal and bacterial keratitis

| Parameters | NT+CAZ+VAN  (n = 7) N (%) | VRC+CAZ+VAN  (n= 16) N (%) | *p-value* |
| --- | --- | --- | --- |
| Sex  Male  Female | 2 (28.6)  5 (71.4) | 7 (43.8)  9 (56.3) | 0.657 |
| Age (years)  median (IQR) | 60.0 (49.50 – 65.0) | 44.50 (32.50 – 66.0) | 0.181 |
| Age mean ± SD | 57.29 ± 12.78 | 46.19 ± 19.29 |  |
| Onset (days) |  |  |  |
| median (IQR) | 18.0 (11.0 – 33.0) | 10.50 (6.0 – 60.0) | 0.720 |
| mean ± SD | 24.0 ± 20.96 | 27.88 ± 32.04 |  |
| Ulcer site |  |  |  |
| No ulcer | 0 (0.0) | 0 (0.0) | 0.366 |
| Central | 6 (85.7) | 10 (62.5) |  |
| Paracentral | 1 (14.3) | 6 (37.5) |  |
| Peripheral | 0 (0.0) | 0 (0.0) |  |
| Ulcer depth |  |  |  |
| Superficial 2/3 of stroma | 4 (57.1) | 13 (81.3) | 0.318 |
| Posterior 1/3 of stroma | 3 (42.9) | 3 (18.8) |  |
| Ulcer size (mm) |  |  |  |
| Median (IQR) | 4.50 (3.50 – 4.75) | 3.50 (3.50 – 4.25) | 0.579 |
| Mean ± SD | 4.36 ± 1.18 | 4.58 ± 2.33 |  |
| Small (≤2mm) | 0 (0.0) | 0 (0.0) | 1.000 |
| Medium (>2 to ≤ 6) | 6 (85.7) | 13 (81.3) |  |
| Large (> 6 mm) | 1 (14.3) | 3 (18.8) |  |
| Infiltrate size |  |  |  |
| ≤ ulcer size | 5 (71.4) | 13 (81.3) | 0.621 |
| > ulcer size | 2 (28.6) | 3 (18.8) |  |
| Hypopyon | 3 (42.9) | 7 (43.8) | 1.000 |
| Abscess | 3 (42.9) | 2 (12.5) | 0.142 |
| BCVA at baseline |  |  |  |
| median (IQR) | 0.002 (0.002 – 0.01) | 0.01 (0.002 – 0.01) | 0.341 |
| mean ± SD | 0.003 ± 0.002 | 0.004 ± 0.002 |  |

Abbreviations: BCVA: best-corrected visual acuity, IQR: interquartile range, SD: standard deviation,

NT+CAZ+VAN: natamycin 5% + fortified ceftazidime 5% + vancomycin 5%, VRC+CAZ+VAN: voriconazole 1% + fortified ceftazidime 5% + vancomycin 5%

**Table S5** Treatment outcomes and responses to empiric antibiotics regimens: MOX, GEN +VAN, and CAZ+VAN in patients with bacterial keratitis

| Outcomes, responses, and complications | MOX  (n = 31)  N (%) | GEN+VAN  (n = 9)  N (%) | CAZ+VAN  (n = 9)  N (%) | *p-value* |
| --- | --- | --- | --- | --- |
| Response to therapy |  |  |  |  |
| Improving | 20 (64.5) | 8 (88.9) | 4 (44.4) | 0.241 |
| Worse | 9 (29.0) | 1 (11.1) | 3 (33.3) |  |
| Not changing | 2 (6.5) | 0 (0.0) | 2 (22.2) |  |
| Ulcer healing | (n = 31) | (n = 7) | (n = 9) |  |
| Not healed | 11 (35.5) | 1 (14.3) | 5 (55.6) | 0.259 |
| Healed | 20 (64.5) | 6 (85.7) | 4 (44.4) |  |
| Intervention |  |  |  |  |
| No | 20 (64.5) | 6 (66.7) | 4 (44.4) | 0.593 |
| Yes | **11 (35.5)** | **3 (33.3)** | **5 (55.6)** |  |
| TPK | 3 (9.7) | 0 (0.0) | 3 (33.3) | 0.136 |
| Treatment changed | 8 (25.8) | 3 (33.3) | 2 (22.2) | 0.909 |
| Time to epithelialization (days) | (n = 20) | (n = 6) | (n = 4) |  |
| Mean ± SD | 32.25 ± 17.60 | 18.50 ± 9.20 | 37.25 ± 19.75 | 0.103 |
| Complications^#^ |  |  |  |  |
| Corneal perforation | 2 (6.5) | 0 (0.0) | 2 (22.2) | 0.297 |
| Corneal melting | 4 (12.9) | 0 (0.0) | 2 (22.2) | 0.388 |
| Persistent epithelial defect | 7 (22.6) | 1 (11.1) | 2 (22.2) | 0.884 |
| Corneal opacity | 19 (61.3) | 8 (88.9) | 3 (33.3) | 0.070 |
| Anterior synechiae/ adherent leukoma | 1 (3.2) | 0 (0.0) | 0 (0.0) | 1.000 |
| Corneal thinning/ descemetocele | 1 (3.2) | 0 (0.0) | 2 (22.2) | 0.165 |
| Post treatment BCVA | (n = 20)  0.10 (0.020 – 0.20) | (n = 6) | (n = 4) |  |
| Median (IQR) |  | 0.01 (0.005 – 0.10) | 0.04 (0.024 – 0.05) | 0.225 |
| Mean ± SD. | 0.152 ± 0.192 | 0.040 ± 0.047 | 0.037 ± 0.017 |  |
| Improvement in BCVA | (n = 20) | (n = 6) | (n = 4) |  |
| Min. – Max. | 0.0 – 0.200 | 0.0 – 0.095 | 0.009 – 0.045 | 0.577 |
| Median (IQR) | 0.045 (0.0 – 0.171) | 0.009 (0.0 – 0.067) | 0.04 (0.02 – 0.05) |  |
| Mean ± SD. | 0.075 ± 0.080 | 0.030 ± 0.041 | 0.032 ± 0.017 |  |

Significant *p*-values are in bold

#: The same patient could have more than one complication

Abbreviations: BCVA: best-corrected visual acuity, IQR: interquartile range, SD: standard deviation,

MOX: moxifloxacin 0.5%, fortified GEN+VAN: gentamicin 1.4% + vancomycin 5%, fortified CAZ+VAN: ceftazidime 5% + vancomycin 5%, TPK: therapeutic penetrating keratoplasty

**Table S6** Treatment outcomes and responses to empiric antifungal regimens: NT, VRC, and NT+VRC in patients with fungal keratitis

| Outcomes, responses,  and complications | NT  (n = 19)  N (%) | VRC  (n = 23)  N (%) | NT+VRC  (n = 9)  N (%) | *p-value* |
| --- | --- | --- | --- | --- |
| Response to therapy |  |  |  |  |
| Improving | 10 (52.6) | 9 (39.1) | 8 (88.9) | 0.093 |
| Worse | 4 (21.1) | 10 (43.5) | 1 (11.1) |  |
| Not changing | 5 (26.3) | 4 (17.4) | 0 (0.0) |  |
| Ulcer healing |  |  |  |  |
| Not healed | 9 (47.4) | 14 (60.9) | 1 (11.1) | **0.036** **0.018^*^** |
| Healed | 10 (52.6) | 9 (39.1) | 8 (88.9) |  |
| Intervention |  |  |  |  |
| No | 10^ab^ (52.6) | 9^b^ (39.1) | 8^a^ (88.9) | **0.034** **0.018^$^** |
| Yes^#^ | **9** ^ab^ **(47.4)** | **14^b^ (60.9)** | **1^a^ (11.1)** |  |
| TPK | 2 (10.5) | 7 (30.4) | 1 (11.1) | 0.234 |
| AC wash/IC/IS injections with  antifungals | 0^a^ (0.0) | 4^ab^ (17.4) | 1^b^ (11.1) | 0.138 |
| Cyanoacrylate corneal gluing | 0 (0.0) | 1 (4.3) | 0 (0.0) | 1.000 |
| Treatment changed | 7 (36.8) | 5 (21.7) | 0 (0.0) | 0.097 |
| Time to epithelialization (days) | (n = 10) | (n = 9) | (n = 8) |  |
| Mean ± SD | 27.60^b^ ± 17.19 | 68.33^a^ ± 21.12 | 63.75^a^ ± 13.44 | **0.001** |
| Complications^#^ |  |  |  |  |
| Corneal perforation | 1 (5.3) | 6 (27.3) | 1 (11.1) | 0.174 |
| Corneal melting | 3 (15.8) | 6 (27.3) | 0 (0.0) | 0.209 |
| Persistent epithelial defect | 6 (31.6) | 4 (18.2) | 0 (0.0) | 0.170 |
| Corneal opacity | 10 (52.6) | 10 (43.4) | 7 (77.7) | 0.268 |
| Anterior synechiae/ adherent leukoma | 1 (5.3) | 1 (4.5) | 3 (33.3) | 0.089 |
| Corneal thinning/ descemetocele/anterior staphyloma | 1 (5.3) | 3 (13.6) | 1 (11.1) | 0.835 |
| Atrophy | 0 (0.0) | 1 (4.5) | 0 (0.0) | 1.000 |
| Endophthalmitis | 0 (0.0) | 1 (4.5) | 0 (0.0) | 1.000 |
| Post-treatment BCVA | (n = 10)  0.033 (0.01 – 0.40) | (n = 9) | (n = 8) |  |
| Median (IQR) |  | 0.01 (0.01 – 0.03) | 0.005 (0.01 – 0.03) | 0.191 |
| Mean ± SD | 0.155 ± 0.218 | 0.034 ± 0.063 | 0.036 ± 0.068 |  |
| Improvement in BCVA |  |  |  |  |
| Min. – Max. | 0.0 – 0.467 | 0.0 – 0.198 | 0.0 – 0.195 | 0.225 |
| Median (IQR) | 0.017 (0.0 – 0.40) | 0.0 (0.0 – 0.004) | 0.0 (0.0 – 0.02) |  |
| Mean ± SD | 0.139 ± 0.207 | 0.026 ± 0.065 | 0.030 ± 0.069 |  |

Significant *p-* values are in bold

The superscripted letters a and b were used to indicate significant differences between groups in a single row. A common letter between groups indicates no significant difference, while the lack of a common letter indicates a significant difference

*: Significant *p-*value for comparing ulcer healing between VRC and NT+VRC

$: Significant *p-*value for comparing interventions between VRC and NT+VRC

#: The same patient could receive more than one intervention or could suffer from more than one complication

Abbreviations: AC: anterior chamber, BCVA: best-corrected visual acuity, IC: intracameral, IS: intrastromal, IQR: interquartile range, SD: standard deviation, NT: natamycin 5%, VRC: voriconazole 1%, NT+VRC: natamycin 5% + voriconazole 1%, TPK: therapeutic penetrating keratoplasty

**Table S7** Treatment outcomes and responses to empiric antifungal and antibiotic regimens: NT+CAZ+VAN and VRC+CAZ+VAN in patients with mixed fungal and bacterial keratitis

| Outcomes, responses,  and complications | (NT+CAZ+VAN)  (n =7)  N (%) | (VRC+CAZ+VAN)  (n =16)  N (%) | *p-value* |
| --- | --- | --- | --- |
| Response to therapy |  |  |  |
| Improving | 0 (0.0) | 8 (50.0) | **0.048** |
| Worse | 4 (57.1) | 6 (37.5) |  |
| Not responding | 3 (42.9) | 2 (12.5) |  |
| Ulcer healing |  |  |  |
| Not healed | 7 (100.0) | 8 (50.0) | 0.052 |
| Healed | 0 (0.0) | 8 (50.0) |  |
| Intervention |  |  |  |
| No | 0 (0.0) | 8 (50.0) | 0.052 |
| Yes | **7 (100.0)** | **8 (50.0)** |  |
| TPK | 2 (28.6) | 5 (31.25) | 1.000 |
| Treatment changed | 5 (71.4) | 3 (18.75) | **0.026** |
| Time to epithelialization (days) | (n = 0) | (n = 8) |  |
| Median (IQR) | – | 55.0 (47.50 – 70.0) | – |
| Mean ± SD | – | 58.13 ± 19.26 |  |
| Complications^#^ |  |  |  |
| Corneal perforation | 1 (14.2) | 2 (12.5) | 1.000 |
| Corneal melting | 3 (42.8) | 6 (37.5) | 1.000 |
| Persistent epithelial defect | 2 (28.5) | 3 (18.75) | 0.621 |
| Corneal opacity | 0 (0.0) | 7 (43.75) | 0.057 |
| Anterior synechiae/ adherent leukoma | 0 (0.0) | 1 (6.25) | 1.000 |
| Corneal thinning/ descemetocele | 2 (28.5) | 0 (0.0) | 0.083 |
| Endophthalmitis | 0 (0.0) | 1 (6.25) | 1.000 |
| Post treatment BCVA | (n = 0) | (n = 8) |  |
| Median (IQR) | – | 0.01 (0.01 – 0.04) | – |
| Mean ± SD. | – | 0.028 ± 0.03 |  |
| Improvement in BCVA | (n = 0) | (n = 8) |  |
| Min. – Max. | – | 0.0 – 0.10 | – |
| Median (IQR) | – | 0.01 (0.0 – 0.04) |  |
| Mean ± SD. | – | 0.023 ± 0.03 |  |

Significant *p*-values are in bold

#: The same patient could suffer from more than one complication

Abbreviations: BCVA: best-corrected visual acuity, IQR: interquartile range, SD: standard deviation

NT+CAZ+VAN: natamycin 5% + fortified ceftazidime 5% + vancomycin 5%, VRC+CAZ+VAN: voriconazole 1% + fortified ceftazidime 5% + vancomycin 5%, TPK: therapeutic penetrating keratoplasty

**3-Supplementary Figures**


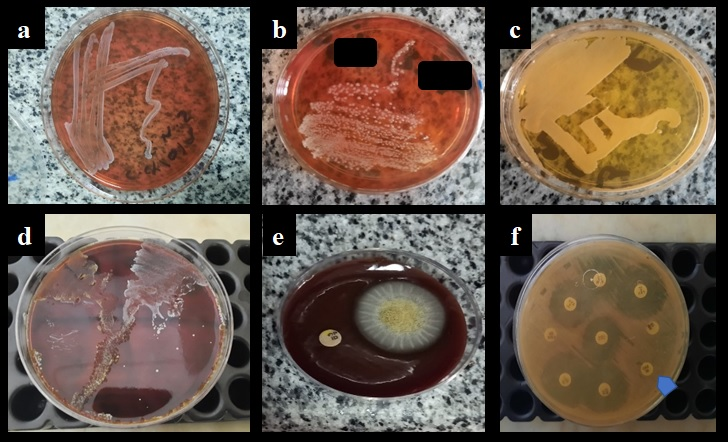


**Fig. S1** Cultivation of corneal scraping samples on different culture media. **(a)** Blood agar showing the growth coagulase-negative staphylococci, **(b)** Mannitol salt agar (MSA) showing pink colonies corresponding to coagulase-negative staphylococci, **(c)** MSA growing golden-yellow-colored colonies of *S. aureus*, **(d)** MacConkey agar showing mucoid growth of Gram-negative bacteria, **(e)** Sabouraud’s Dextrose Agar growing filamentous fungi, **(f)** Muller-Hinton agar inoculated with *P. aeruginosa* showing susceptibility to gentamicin, tobramycin, and levofloxacin, and resistance to ceftazidime (blue arrow), using the disc diffusion method


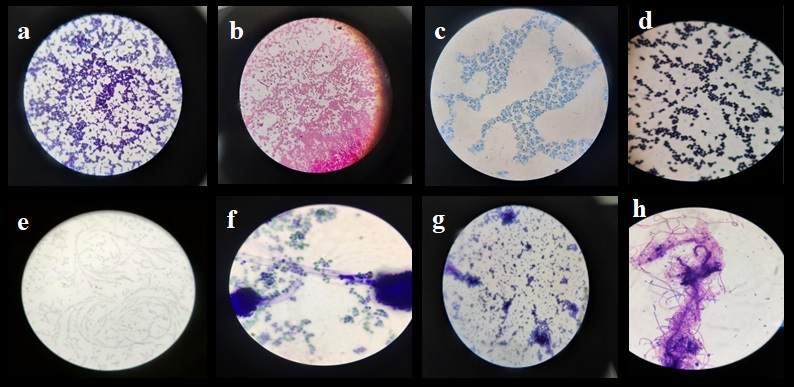


**Fig. S2** Microscopic identification of the causative microorganisms of microbial keratitis from cultured corneal scrapings under the microscope (oil lens magnification x 100). **(a)** Coagulase-negative staphylococci showing clusters in Gram-stained mount, **(b)** Gram-negative bacilli of *P. aeruginosa* in Gram-stained mount, **(c)** *Candida* spp*.* showing budding cells in Giemsa-stained mount, **(d)** *Candida* spp*.* showing budding cells in Gram-stained mount, **(e)** branching hyphae of filamentous fungi in direct KOH mount, **(f)** *Aspergillus* spp*.* showing spores and hyphae in Giemsa-stained mount, **(g)** *Candida* spp. mixed with Gram-positive coagulase-negative staphylococci (CoNS) showing budding cells of *Candida* with CoNS clusters in Gram-stained mount, **(h)** filamentous fungi mixed with Gram-positive *Streptococci* spp. isolated from mixed fungal and bacterial keratitis


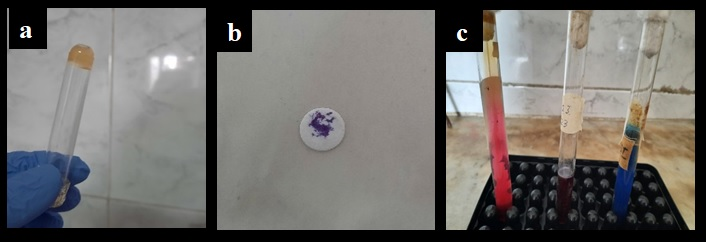


**Fig. S3** Biochemical tests performed for the identification of isolated microorganisms from corneal scrapings of diagnosed patients with bacterial keratitis. **(a)** Positive coagulase test in identified MRSA, **(b)** positive oxidase test in identified *P. aeruginosa*, **(c)** triple sugar iron agar test, motility indole ornithine, and citrate test (from left to right) in an identified isolate of *P. aeruginosa*

**REFERENCES**

52. Ratprasatporn N, Wittayalertpanya S, Khemsri W, et al. (2019) Stability and sterility of extemporaneously prepared nonpreserved cefazolin, ceftazidime, vancomycin, amphotericin B, and methylprednisolone eye drops. Cornea 38: 1017-1022. doi: 10.1097/ico.0000000000001992

53. Dupuis A, Tournier N, Le Moal G, et al. (2009) Preparation and stability of voriconazole eye drop solution. Antimicrob Agents Chemother 53: 798-799. doi: 10.1128/aac.01126-08

54. Khan ZA, Siddiqui MF, Park S (2019) Current and emerging methods of antibiotic susceptibility testing. Diagnostics (Basel) 9. doi: 10.3390/diagnostics9020049

55. CLSI. Method for antifungal disk diffusion susceptibility testing of yeasts. 3rd ed.CLSI guideline M44. Clinical and Laboratory Standards Institute, 950 West Valley Road, Suite 2500, Wayne, Pennsylvania 19087 USA, 2018.

56. Antimicrobial resistant phenotype definitions <https://www.cdc.gov/nhsn/pdfs/ps-analysis-resources/aur/ar-phenotype-definitions-508.pdf>. Accessed 18 April 2023
